# Control of Gene Expression by RNA Binding Protein Action on Alternative Translation Initiation Sites

**DOI:** 10.1371/journal.pcbi.1005198

**Published:** 2016-12-06

**Authors:** Angela Re, Levi Waldron, Alessandro Quattrone

**Affiliations:** 1 Laboratory of Translational Genomics, Centre for Integrative Biology, University of Trento, Polo Scientifico e Tecnologico Fabio Ferrari, Trento, Italy; 2 City University of New York Graduate School of Public Health and Health Policy, New York, New York, United States of America; National Center for Biotechnology Information (NCBI), UNITED STATES

## Abstract

Transcript levels do not faithfully predict protein levels, due to post-transcriptional regulation of gene expression mediated by RNA binding proteins (RBPs) and non-coding RNAs. We developed a multivariate linear regression model integrating RBP levels and predicted RBP-mRNA regulatory interactions from matched transcript and protein datasets. RBPs significantly improved the accuracy in predicting protein abundance of a portion of the total modeled mRNAs in three panels of tissues and cells and for different methods employed in the detection of mRNA and protein. The presence of upstream translation initiation sites (uTISs) at the mRNA 5’ untranslated regions was strongly associated with improvement in predictive accuracy. On the basis of these observations, we propose that the recently discovered widespread uTISs in the human genome can be a previously unappreciated substrate of translational control mediated by RBPs.

## Author Summary

Gene expression is a dynamic program by which the information stored in the genome is rendered functional by production and degradation of two types of macromolecules, RNAs and proteins. mRNAs are templates for proteins; therefore we expect correspondence between quantities of mRNAs and proteins. Genome-wide studies instead indicate a marked discrepancy between them, when considering their steady-state levels or their variations across different conditions. We employed linear regression approaches with paired mRNA/protein datasets in order to develop a model predicting the protein level of a gene from both the mRNA level and the protein levels of RBPs inferred to bind the mRNA untranslated regions. The results of our analyses restricted the utility of RBPs to improve accuracy of predicted protein abundance to a small fraction of the total modelled genes, and identified a novel association of the improvement induced by RBPs with the presence of upstream translation sites. This finding suggests a new avenue of experimental studies aimed at exploring the hypothesis that RBPs could influence protein abundance by changing the preference for certain translation initiation sites.

## Introduction

High throughput technologies such as RNA-sequencing (RNA-seq) and mass-spectrometry-based protein analyses provide transcriptomic and proteomic profiles, which are the basis to draft a comprehensive picture of gene expression regulation [1],[2],[3].

Several studies have reported a lack of concordance between transcriptome and the proteome profiles [3],[4],[5],[6],[7], both at the steady state [8],[9],[10] and dynamically [11],[12],[13]. Even though this phenomenon is partially accounted for by technical factors such as noise [14], biased detection [15] and limited and variable coverage of mRNA and protein measurements [16], the discrepancy is so considerable that undoubtedly it implies an unresolved complexity in the regulation of gene expression downstream of transcription. Several studies have sought to examine the extent to which specific levels of regulation contribute to determine protein abundance at the steady state [17],[13],[8]. It was initially estimated that in mouse fibroblasts transcription explains 34% of variance in protein abundance, mRNA degradation 6%, translation 55% and protein degradation 5% [8]. Employing additional statistical efforts to account for the influence of measurement error on mRNA/protein correlation, recent studies proposed a correction of the initial estimates and brought back the role of translation to 30% [18]. Several studies highlighted the strong influence of translation on differential protein abundance during dynamic responses [19], [20], [21], [22].

The regulatory mechanisms by which the various post-transcriptional processes exert their effects on protein abundance are not well understood. Regulatory features associated with these processes have been identified not only in the coding regions but also in the 5’ and 3’ untranslated regions (UTRs) of mRNAs in multiple species [23],[24]. After their synthesis, processing, and export to the cytoplasm, mRNAs are broadly engaged in two activities: they may serve as templates for translation or as substrates for degradation pathways. Translational control, principally involving the initiation stage, can occur on a global basis by changes in the amounts and activation state of components of the translational machinery: translation factors [25], tRNAs [26] and ribosomes [27],[28]. Transcript-specific control of translation is less understood. The mechanisms of selective translation through recognition of target mRNAs by trans-acting factors, such as non-coding RNAs [28],[29],[30] and RNA-binding proteins (RBPs) [31],[32], are still subject of investigation [33],[34],[35],[36],[37], and are known only in a limited number of cases [38],[39],[40],[27],[41],[42].

Here, we developed a model of post-transcriptional control of gene expression by using multivariate linear regression to estimate protein levels from transcript levels. The model is empirically developed from two types of primary data: quantitative transcriptome assays matched with proteome assays, and post-transcriptional regulatory annotations of mRNA untranslated regions (UTRs) obtained by scanning for occurrences of in vitro experimentally determined RBP binding sites [31]. Including RBP levels and binding sites resulted in a statistically significant improvement of accuracy in protein abundance estimates of a fraction of the total modeled mRNAs in three panels of tissues and cells. We showed this improvement to be associated with the presence of upstream translation initiation sites (uTISs). This observation suggests the possibility that RBP could influence protein abundance by modulating alternative translation initiation, a mechanism of translational control still not experimentally described.

## Results

### Developing the model

To devise a model of protein levels from transcript levels including a quantitative description of the contribution of RBP-mediated post-transcriptional control, we selected three data panels consisting of matched transcript and protein profiles: twelve normal human tissues [43], 59 cancer cell lines (the NCI-60 panel) [44], and 87 colorectal cancer tissues (the CPTAC CRC panel) [45]. The normal tissue panel contains the widest physiological variability, therefore it was used for determining model predictiveness. The NCI-60 and CPTAC CRC panels were used to show repeatability of the major findings in independent panels, and to assess cross-panel transferability of protein abundance models.

The depth of proteome coverage in the normal tissue panel was substantially lower than of the transcriptome (**Table A in S1 Text**), confirming previous reports [46]. We avoided genes whose transcripts and proteins were not reliably measured in a substantial number of samples in each panel (**S1 Fig**). This filtering resulted in the selection of more highly abundant genes than the overall pool at either the mRNA or protein level (**S2 Fig**). This effect was expected, considering the low frequency at which lowly abundant peptides could be selected for peptide sequence analysis and subsequent protein quantification. Filtering for adequately measured proteins introduced a bias in the genes we were able to study, highlighted by depletion and enrichment of several Gene Ontology (GO) categories (**S3 Fig**). The NCI-60 and CPTAC CRC panels also showed partial proteome coverages (**Table A in S1 Text**, **S1 Fig**), and consequent biases (**S2 Fig**, **S3 Fig**).

When measuring gene expression, multiple biological and technical factors can interact to produce the variability in average mRNA/protein levels, which we observed across the samples of each panel (**S4 Fig, S5 Fig**). To eliminate the possibility that average protein levels could help in predicting protein abundance (**S6 Fig**), mRNA and protein data were mean-centred per sample in each panel (**Supporting Information**). No sample turned out to be systematically associated with outlier measurements in any of the three panels (**Supporting Information**, **S7 Fig**).

We used RNA-binding motifs in linear regression modeling to infer models of RBP post-transcriptional regulation for all genes where transcripts, proteins, and RBPs were measured in a sufficient number of samples in a panel. The compendium of RNA-binding motifs was derived for 85 human RBPs by RNAcompete [29], an in vitro method for rapid and systematic analysis of RNA sequence preferences of RBPs shown to be predictive of in vivo binding [47]. We scanned the 5’ and 3’ UTRs of the mRNAs to identify sequences matching to the RNA-binding motifs, and detected RBP binding sites for 50 RBPs within the 5’ and 3’ UTRs of the 1,109 genes modeled in the normal tissue panel (q < 0.20). For genes modeled in the NCI-60 panel we identified binding sites for 40 RBPs on 1,327 mRNAs; in the CPTAC CRC panel for 66 RBPs on 1,825 mRNAs. The inferred RBP-mRNA interactions confirmed the previously reported tendency of multiple mRNAs to be regulated by multiple RBPs [47],[48],[49], with the number of RBPs per mRNA ranging from 1 to 38 based on inferred RBP binding sites in mRNA UTRs. This observation was independent of the stringency in statistical significance used for predicting RBP binding sites (**S8 Fig**).

We assessed the accuracy of the RBP-inclusive models to predict the protein abundance of modelled mRNAs by cross-validation and cross-panel validation. Finally, the relevance of RBPs in transcript/protein coupling was tested for association with regulatory features of the modelled mRNAs.

#### RNA binding proteins improve prediction of protein levels in normal tissues

**Fig 1:**
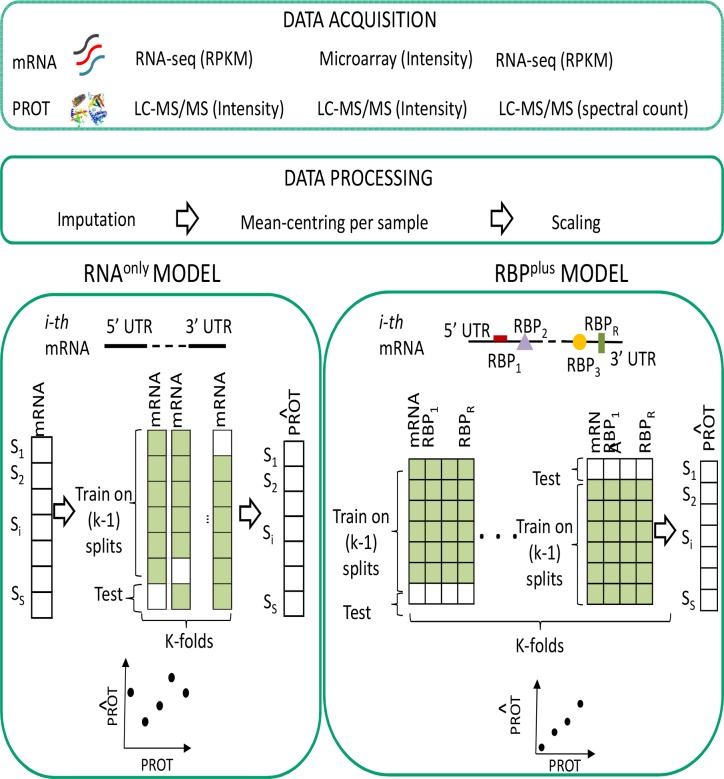
Data modelling workflow. Primary data consist of three panels of quantitative transcriptome assays matched with proteome assays. Panels differ by cellular state and technological platforms for quantification of transcript and protein abundance. Data modelling is performed in parallel in the three panels. For each mRNA, we compare the accuracy of two models to predict abundance of the corresponding protein: a basic model (RNA^only^) that predicts level of the protein from its mRNA level only, and a RBP-inclusive model (RBP^plus^) containing additional candidate predictors defined by protein levels of the RBPs which were inferred by sequence specificity to bind the mRNA UTRs. Data used in each type of model are visualized with matrices where samples (S) are shown by row and predictors (mRNA, RBP protein levels) by column. Accuracy of predicted protein abundance was assessed by k-fold (k = 5) cross-validation.

**Fig 2:**
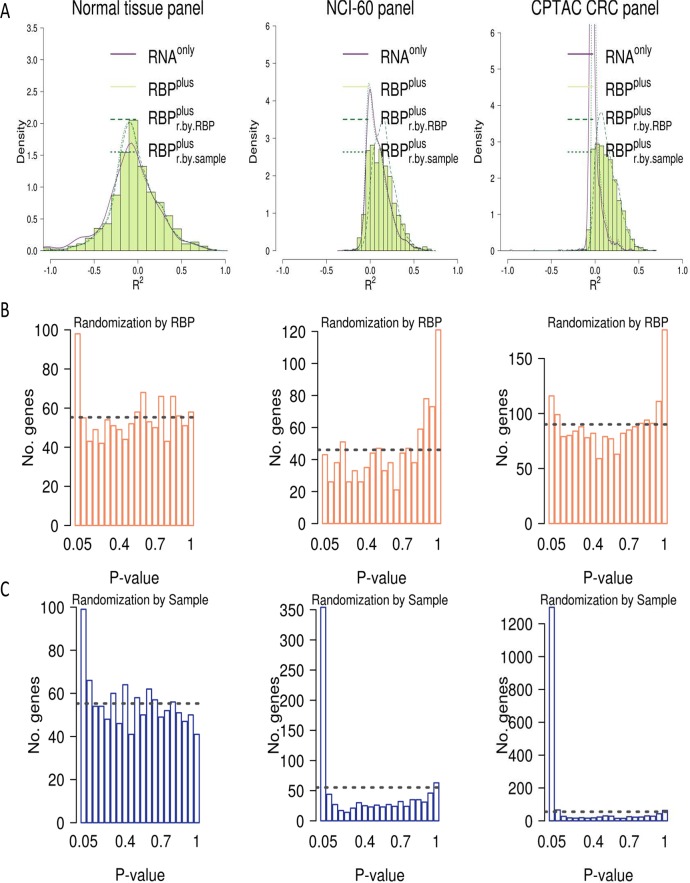
Inferred RBP-mRNA interactions improve accuracy in predicting protein abundance of a portion of the total modeled mRNAs in three panels of tissues and cell lines. While RBP^plus^ models improve accuracy (R^2^) in predicted protein abundance over RNA^only^ models, improvements attained by RBPs were not distinguishable from those by randomly sampled proteins, for the majority of genes considered in the three panels. The proportion of genes where actual RBPs produced higher accuracy than random protein predictors (q < 0.05) increases from 0.65% in the NCI-60 panel to 4.2% in the normal tissue panel. **(A)** Distribution of R^2^ coefficients for the actual RNA^only^ and RBP^plus^ models as well as for the RBP^plus^ models randomized either by permuting sample labels (RBP^plus^_r.by.sample_) or by randomly sampling proteins in place of actual RBPs (RBP^plus^_r.by.RBP_). **(B)** Histogram of statistical significance estimates for the RBP^plus^ models which were obtained randomizing the actual RBP^plus^ models by randomly sampling proteins. **(C)** Histogram of statistical significance estimates for the RBP^plus^ models which were obtained randomizing the actual RBP^plus^ models by permuting sample labels. Dashed line corresponds to the number of genes expected in each bin under the assumption of a uniform distribution.

The majority of genes in the normal tissue panel exhibited low correlation between transcriptome and proteome profiles (Spearman’s correlation coefficient < 0.42 for 75% of genes, median = 0.20). To estimate the impact of RBP-based post-transcriptional control in this discordance, we built two types of models for each considered gene: a baseline model predicting protein level from only the corresponding mRNA level (RNA^only^) in a simple linear regression model, and a RBP-inclusive multiple linear regression model (RBP^plus^) which predicts the protein level of a gene from both mRNA level and protein levels of RBPs inferred to bind the mRNA UTRs (**Fig 1**). For each considered gene, we used simple linear regression in the RNA^only^ model and maximum penalized likelihood regression for the RBP^plus^ model, with Ridge penalty [48] applied to RBP covariates but with mRNA unpenalized. With this approach, in case of uninformative RBP covariates, we expect the RBP^plus^ model to converge to the RNA^only^ one. We fitted as many RNA^only^ and RBP^plus^ models as considered mRNAs. A network view of RBP-mRNA interaction network derived from the RBP^plus^ models is provided in **S9 Fig**. Prediction accuracy of RNA^only^ and RBP^plus^ models was quantified by absolute R^2^ coefficient of determination, and evaluated by 5-fold cross-validation, with training performed entirely on training samples, and only predictions for held-out test samples used for accuracy estimation. Note that the effectiveness of cross-validation scheme at avoiding inflated model accuracy was confirmed when tissues labels for proteins being predicted were permuted, resulting in accuracies centred on the expected null value (**Fig 2A**). The RBP^plus^ model produced an improvement in the accuracy of predicted protein levels relative to the RNA^only^ model (Wilcoxon signed-rank test, p = 3 10^−5^). This improvement was statistically significant for different false discovery rate thresholds for predicting RBP binding sites (**S10 Fig**). Further, we explored whether penalized regression with Least Absolute Shrinkage and Selection Operator (LASSO) [49] penalty, which operates variable selection, could also capture improvement in protein predictive accuracy of the RBP^plus^ over the RNA^only^ model. For this purpose, we used the same procedure for penalized regression replacing Ridge with LASSO penalty to fit the RBP^plus^ models, and tested the differences in rank of protein predictive accuracies for the LASSO penalized RBP^plus^ model and the RNA^only^ model (Wilcoxon signed-rank test). We found that the RBP^plus^ models fitted by LASSO penalty achieved statistically significantly higher predictive accuracy relative to the RNA^only^ models (p = 6 10^−11^, **S11 Fig**). Further, the RBP^plus^ models fitted by LASSO and Ridge penalty were found to perform almost equivalently (Wilcoxon signed-rank test, p = 0.01, **S12A Fig**). As expected, the number of predictors in the RBP^plus^ model was much lower when models were fitted by LASSO penalty instead of by Ridge penalty (**S12B Fig**), with an average fraction of selected predictors per gene of ~10%. The genes where selected predictors reduced to just the mRNA of the modelled gene were found to represent ~33% of the modelled genes.

**Fig 3:**
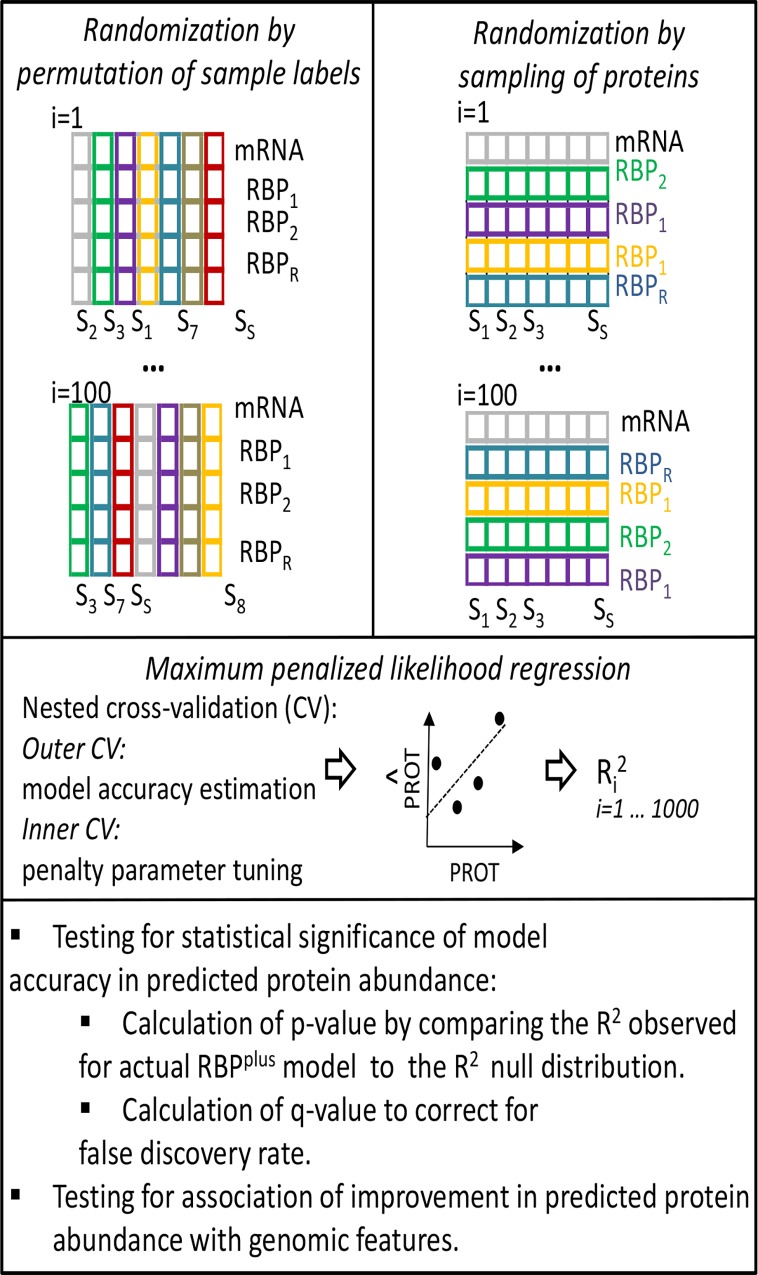
Scheme of tests assessing statistical significance of the accuracy of RBP^plus^ model to predict protein abundance and association of accuracy with genomic features. For each gene, 1000 randomized versions of the RBP^plus^ model were obtained either by permuting the RBP protein levels across samples (left side), or by randomly sampling a number of protein predictors equal to the number of actual RBPs inferred to bind the mRNA UTRs (right side). The two randomization tests were run in parallel for each gene. Each randomized model was fitted with Ridge penalized linear regression using nested cross-validation (CV). In the nested cross-validation scheme, test samples are held out for accuracy estimation in the outer layer of CV, and penalty parameters are tuned in the inner layer of CV within training samples only. The p-value of the RBP^plus^ model of each gene was defined by the probability of sampling a R^2^ value from the empirical null distribution higher than the R^2^ observed for the actual RBP^plus^ model. False Discovery Rate was estimated by Storey’s q-value method.

To complete our assessment of RBP^plus^ model predictive accuracies, we still need to estimate the extent to which the contribution of inferred RBPs to improve protein predictions departs from the contribution of random predictors. For this purpose, we implemented empirical randomization-based tests to determine whether the prediction accuracy obtained by the RBP^plus^ model of each individual gene was statistically significantly better than expected for randomized RBP^plus^ models of the gene. For each considered gene, we developed 1000 randomized versions of the RBP^plus^ model by 1) randomly sampling a number of protein predictors equal to the number of actual RBPs inferred to bind the mRNA UTRs, and 2) by permuting the RBP protein levels across samples (**Fig 3**). The RNA^only^ model was not randomized. Each randomized model was fit following the same implementation of maximum Ridge penalized likelihood regression by nested cross-validation used for the actual RBP^plus^ models. For each gene, we computed raw p-values as the maximum of 1) the proportion of accuracies attained by the randomized models higher than the accuracy of the actual RBP^plus^ model of the gene, or 2) 1/1000 (1000 being the number of permutations) if no permutation accuracies exceeded the accuracy of the actual RBP^plus^ model. Correction for False Discovery Rate was then performed using Storey’s q-value method [50]. Improvements in predictive accuracy attained by RBPs were not distinguishable from improvements attained using randomly sampled proteins as covariates, for the majority of genes considered in the three panels (**Fig 2A**). Statistical testing based on protein randomization confirmed that RBPs were not generally useful to improve protein predictions but in ~9% of the genes considered in the normal tissues (**Fig 2B**). Collinearity between predictors is a probable reason for the failure of inferred RBPs to improve protein predictions relative to randomly sampled predictors in many models. We performed over-/under-representation analysis of GO themes in the genes where the RBP^plus^ model was nominally significant (p < 0.05), using uncorrected p-values for GO analysis to reduce the false negative rate of a stricter FDR-based threshold. The most highly overrepresented Gene Ontology categories in genes with informative RBP^plus^ models were related to mRNA processing and translation, processes already known to be particularly prone to post-transcriptional control (**S13 Fig**). As expected, when we randomized the RBP^plus^ models by permuting RBP protein levels across samples, these models were equivalent to the RNA^only^ models (**Fig 2A**). Statistical testing confirmed that the RBP^plus^ model achieved better predictive accuracy than expected for RBP^plus^ models randomized by sample permutation (*p* < 0.05) in 8.9% of the genes considered in normal tissues (**Fig 2C**). After False Discovery Rate correction, the RBP^plus^ model was confirmed to improve accuracy of predicted protein abundance in 4.2% and 11% of considered genes when, respectively, randomizing the RBP^plus^ model by randomly sampling protein predictors or by permuting samples (Storey’s q < 0.05).

Normalization by mean-centering mRNA and protein data in each sample ensured that average protein levels could not be predictive. Furthermore, the number of proteins in the RBP^plus^ model was uncorrelated to predictive accuracy (**S14 Fig**).

In summary, this analysis allowed identification of a small portion of annotated genes where the contribution of RBPs helped predict protein levels relative to randomly selected protein predictors.

#### Extension of the RNA binding protein improvement of protein predictability to cancer cells and tissues

As already said, we additionally analyzed matched transcriptomic and proteomic profiles from the NCI-60 cell lines and the CPTAC CRC tissues to assess generalizability of these findings and transferability of the models. The NCI-60 panel of the US National Cancer Institute’s Developmental Therapeutics Program [44] encompasses matched transcript and protein data across 59 cancer cell lines. The CPTAC CRC panel is the result of a proteomic analysis which the Clinical Proteomic Tumour Analysis Consortium (CPTAC) performed on 87 colorectal (CRC) tumour samples for which matched transcriptomic data are available in The Cancer Genome Atlas [45]. It is worth noting that the three panels (normal tissue, NCI-60 and CPTAC CRC) are heterogeneous in terms of technological platforms, quantification methods and biological state, since the second two panels are composed by cancer cells and tissues (**Fig 1**). Filtering on the basis of adequately measured genes and inference of RBP binding sites in mRNA UTRs were performed in full similarity to the normal tissue panel. In analogy to the normal tissue panel, RNA^only^ and RBP^plus^ models for each mRNA/protein pair were fitted using mRNA level as an unpenalized covariate (so that RNA^only^ model is simple linear regression), and RBP protein levels as penalized covariates. Statistical significance of the protein prediction accuracy obtained by the RBP^plus^ model for each considered gene was evaluated by the empirical randomization tests previously described (**Fig 3**). RBP^plus^ models were found to improve protein predictive accuracy with respect to RNA^only^ models, as shown in **Fig 2A** (Wilcoxon signed-rank test, p_NCI-60_ = 4 10^−10^, p_CPTAC CRC_ < 10^−16^). Using the more stringent threshold of 5% to the FDR on RBP binding site predictions, this improvement was confirmed in the CPTAC CRC panel (Wilcoxon signed-rank test, p < 10^−16^) but not in the NCI-60 panel (Wilcoxon signed-rank test, p = 0.1), as shown in **S10 Fig**. Additionally, RBP^plus^ models fitted by LASSO penalty produced better predictive accuracy relative to the RNA^only^ models (Wilcoxon signed-rank test, p_NCI-60_ = 4 10^−4^, p_CPTAC CRC_ < 10^−16^, **S11 Fig**), and Ridge penalized RBP^plus^ models obtained better predictive accuracy than LASSO penalized RBP^plus^ models only in the CPTAC CRC panel (**S12 Fig**). Empirical randomization statistical testing showed that the genes where RBPs improved predictive accuracy (p < 0.05 by randomization of protein predictors) represented 4.7% and 6.4% of the considered genes in the NCI-60 and CPTAC CRC panels, respectively (**Fig 2C**). The RBP-mRNA interactions improved the predicted protein abundance in 0.65% and 1.3% of the genes considered in the NCI-60 and CPTAC CRC panels when randomizing the RBP^plus^ model by randomly sampling protein predictors, and in 21% and 70% of the genes in the NCI-60 and CPTAC CRC panels when permuting samples (q < 0.05). The Gene Ontology overrepresentation profile of genes with nominal p < 0.05 in the NCI-60 and CPTAC CRC panels were more similar to each other than to the normal tissue panel (**S13 Fig**). Indeed, while translation and mRNA processing emerged as common themes, other categories related to protein folding, protein targeting to subcellular localization and cell cycle emerged just in these two additional panels. Collectively, from our analysis of these three panels, we conclude that RBPs were able to improve the accuracy in predicting the protein levels in a small fraction of the genes studied (**S1 File**).

At this point, we assessed model transferability using a cross-panel independent validation scheme. Since the proteome coverages in the three panels were different (**S15 Fig**), RBP^plus^ models were trained using only the RBPs profiled in both the training and test panels. We estimated model transferability computing Spearman’s correlation coefficient of protein predictive accuracies between the RBP^plus^ models trained in a chosen panel and the RBP^plus^ models trained in each of the other two panels. Correlation reached statistical significance, ranging from 0.2 to 0.5 depending of the combination of training/test panels (**S16 Fig**). We noticed that better correlation were observed when RBP^plus^ models were trained in the NCI-60 and CPTAC CRC panels and transferred to the normal tissue panel, possibly due to the limitations of training accurate models in only 12 samples of the normal tissue panel.

### Alternative translation initiation sites are associated with the ability of RNA binding proteins to improve accuracy of proteome prediction from the transcriptome

**Fig 4:**
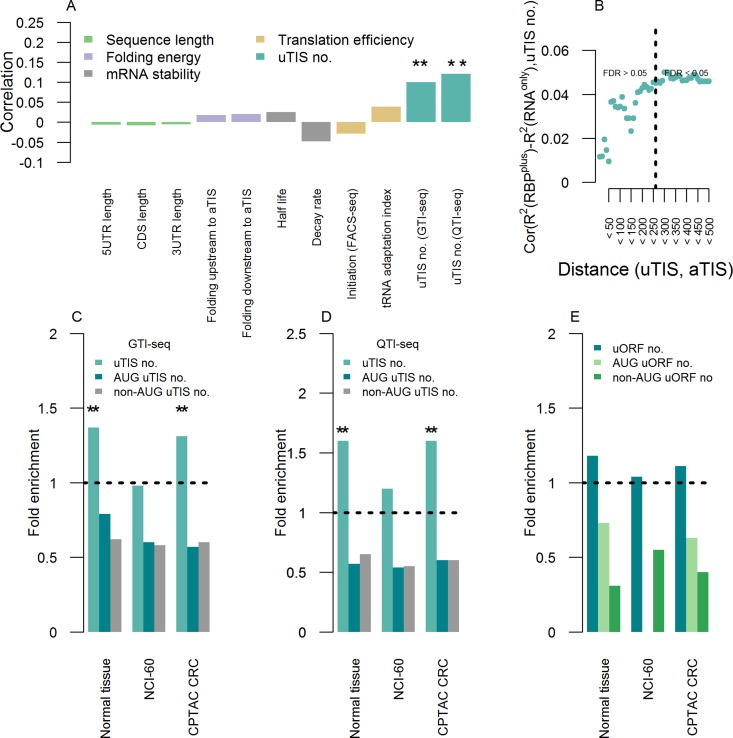
Upstream translation initiation as a prominent feature of the improved predictability of protein levels from transcript levels by RBP^plus^ models. **(A)** Spearman’s correlation coefficient of the improvement in accuracy of predicted protein abundance obtained by the RBP^plus^ model relative to the RNA^only^ one (R^2^_RBP_^plus^—R^2^_RNA_^only^) with several mRNA features. Different colours denote features pertaining to the length of annotated mRNA UTRs and CDS, mRNA folding, mRNA stability, transòlation efficiency and alternative translation by upstream Translation Initiation Sites (uTISs). Analysis is conducted in the panel of human normal tissues. Stars denote statistical significance of correlation (** stands for p < 0.01). **(B)** Spearman’s correlation coefficient between improvement in accuracy of predicted protein abundance and number of uTISs localized at increasing distance upstream to the annotated TIS. Dashed line indicates the distance at which correlation becomes statistically significant. **(C)** uTIS-containing genes are overrepresented in the genes where RBPs improve accuracy of predicted protein abundance relative to the genes where RBPs do not. Shown is the fold enrichment observed for each panel. Stars denote Fisher’s test statistical significance (** stands for p < 0.01). This analysis is based on the uTIS map acquired by GTI-seq in HEK293 cells. **(D)** Overrepresentation is robust to the technological platform for mapping uTISs (QTI-seq in HEK293 cells). **(E)** The association between improvement in predictive accuracy and number of uTISs does not depend on uORFs. uORF-containing genes are not overrepresented in the genes where RBPs are informative relative to the genes where RBPs are not.

We then explored the features associated with the improvement in accuracy of protein abundance prediction achieved by the RBP^plus^ model over the RNA^only^ one, as quantified by the difference in their R^2^ values (R^2^_RBP_^plus^–R^2^_RNA_^only^). For this purpose, we analysed the association of the improvement in predictive accuracy with the major gene-specific sequence and structure annotations of the genes modelled in the normal tissue panel. We considered annotations which have been associated with post-transcriptional regulation of protein abundance [17],[10],[51], and which can be loosely classified by their demonstrated impact mostly on transcript stability and/or translation efficiency (**Table 1**). Spearman’s correlation with most of the tested characteristics was very low (**Table 1**). Interestingly, the only statistically significant correlation was observed between the improvement in accuracy of predicted protein abundance and the number of upstream Translation Initiation Sites (uTISs), as shown in **Fig 4A**.

**Table 1:** Post-transcriptional features quantified in modeled genes.

mRNA annotation	Spearman's correlation	p-value	Data source

5UTR length	-0.006	0.8	The human genome browser at UCSC. Genome Res 2002.
CDS length	-0.008	0.8	The human genome browser at UCSC. Genome Res 2002.
3UTR length	-0.005	0.9	The human genome browser at UCSC. Genome Res 2002.
Folding energy upstream to aTIS	0.02	0.6	The Vienna RNA websuite. Nucleic Acids Res 2008.
Folding energy downstream to aTIS	0.02	0.6	The Vienna RNA websuite. Nucleic Acids Res 2008.
Half life	0.03	0.4	Conserved principles of mammalian transcriptional regulation revealed by RNA half-life. Nucleic Acids Res 2009.
Decay rate	-0.05	0.1	Decay rates of human mRNAs: correlation with functional characteristics and sequence attributes. Genome Res 2003.
Initiation (FACS-seq)	-0.03	0.4	Quantitative analysis of mammalian translation initiation sites by FACS-seq. Mol Syst Biol 2014.
tRNA adaptation index	0.04	0.1	Solving the riddle of codon usage preferences: a test for translational selection. Nucleic Acids Res 2004.
uTIS no. (GTI-seq)	0.10	2 10^−3^	Global mapping of translation initiation sites in mammalian cells at single-nucleotide resolution. Proc Natl Acad Sci U S A. 2012.
uTIS no. (QTI-seq)	0.12	4 10^−5^	Quantitative profiling of initiating ribosomes in vivo. Nat Methods. 2015.

Spearman’s correlation coefficients of the improvement in prediction accuracy obtained by the RBP^plus^ model relative the RNA^only^ model with several mRNA features are reported along with corresponding p-values and data source.

#### Association with upstream translation initiation sites

Upstream translation initiation sites are probed transcriptome-wide by a recently developed high throughput method, Global Translation Initiation sequencing (GTI-seq, [52]). The number of uTISs in the 5’ UTR of a modelled mRNA correlated with the improvement in accuracy of predicted protein abundance (Spearman’s ρ = 0.1, p = 2 10^−3^).

We next explored the relationship between improvement in accuracy of predicted protein abundance with the distance of uTISs from the annotated TISs (aTISs) of modelled mRNAs. For this purpose, we analysed uTIS spatial distribution along the 5’ UTRs of modelled mRNAs and found that uTISs in close proximity to aTISs were rare, with ~15% of mRNAs harbouring at least an uTISs within 50 bases upstream to the aTIS, and tended to locate at an average distance of ~120 nts from the aTISs of modelled mRNAs. The correlation between improvement in predictive accuracy and the number of uTISs increased with distance between uTISs and aTISs of modelled mRNAs (Spearman’s ρ, p < 0.05). As a consequence of uTIS spatial distribution, correlation was scarcely detectable in close proximity to aTISs, and strengthened with distance of uTISs from the aTISs of modelled mRNAs until reaching statistically significant correlation from 250 bases and further (**Fig 4B**).

Importantly, we alternatively confirmed the association between improvement in accuracy of predicted protein abundance and number of uTISs from enrichment of uTIS-containing genes among genes with nominally significant improvement by RBP^plus^ models (p<0.05). This association was significant (p = 2 10^−3^, Fisher’s Exact Test) when we used both canonical and non-canonical uTISs, but not when we used only canonical or only non-canonical uTISs (**Fig 4C**).

We previously mentioned the lack of correlation between improvement in protein predictive accuracy and translation initiation efficiency at the annotated TISs of modelled mRNAs (Spearman’s ρ = -0.03, p = 0.4). Estimates of translation initiation efficiency are obtained by a recent method combining fluorescence-activated cell sorting and high-throughput DNA sequencing (FACS-seq) to quantitate the efficiency of recognition for all possible TIS sequences using ATG start codons [52]. To confirm this result, we extracted features indicative of optimal efficiency in translation initiation from the sequences encompassing the aTISs of modelled mRNAs, and assessed the enrichment/depletion of genes, where RBP^plus^ models improved protein predictions, for highly translationally efficient genes. We considered: (i) the Kozak sequence GCCRCCAUGG (purine, R = A or G) [53], (ii) the -3R and +4G positions which, in particular, are deemed to be the first and second most important bases for efficient translation initiation (+1 denotes the first base of the start codon) and, (iii) the TIS motif (RYMRMVAUGGC) derived from the FACS-seq estimates of translation initiation efficiency. We used separately each type of sequence pattern to define the genes associated with optimal translation initiation efficiency. The genes where RBPs achieved improvements in prediction accuracy over the RNA^only^ model were not found to be enriched or depleted in any of the sequence patterns considered (Fisher’s Exact Test, p > 0.05). Therefore, our results indicate that the improvement in accuracy of predicted protein abundance does not correlate with aTIS efficiency.

#### Lack of association with uORFs

Since uTIS discovery in single loci, efforts have focused on elucidating the molecular effects of a specific subtype of uTISs, the uTIS initiated Open Reading Frames (uORFs). A proposed definition of uORF is: (i) an uTIS out-of-frame at the 5’ UTR, with a stop-codon, in the same frame, downstream of it, and with a minimal length of nine nucleotides, (ii) an uTIS in-frame at the 5’UTR with a stop codon in frame after the main stop codon or before the main start codon. The canonical function of uORFs is to attenuate translation of the primary downstream ORFs [54],[66],[55]. It has hitherto remained largely unknown whether uORFs encode polypeptides that could execute cellular functions [56],[69]. We explored the association of improvement in accuracy of predicted protein abundance with the number of uORFs, and found no statistically significant association (**Fig 4E**).

#### Robustness of association with number of uTISs

We have previously shown that, albeit limited in scope, the predictive value of RBPs was present also in the panels of colorectal cancer samples and NCI-60 cell lines. We assessed the association between improvement in accuracy of predicted protein abundance and number of uTISs in these two panels as well, by Fisher’s Exact Test (**Fig 4C**). The association was confirmed in the colorectal cancer samples (p = 9 10^−3^) but not in the NCI-60 panel of cancer cell lines (p = 0.61).

The uTIS mapping [57] used here was acquired by GTI-seq. We checked that the association between improvement in predictive accuracy and number of uTISs was independent of the technology defining uTISs. For this purpose, we interrogated an independent dataset [58] where TIS positions were systematically profiled by another recently developed technique, Quantitative Translation Initiation sequencing (QTI-seq), which has been reported to identify fewer total TISs than GTI-seq. Similarly to the GTI-seq-based uTIs in the normal tissue panel, we used Fisher’s Exact Test assessed whether genes, where the accuracy in predicted protein abundance was improved by RBPs, were enriched in genes containing uTISs defined by QTI-seq in each of the three panels. The tests reached statistical significance in the normal tissue panel (p = 4 10^−5^) and in the colorectal cancer samples (p = 6 10^−6^) but not in the NCI-60 panel (p = 0.24) (**Fig 4D**). In summary, the association of the improvement in accuracy of predicted protein abundance with the number of uTISs was robust to the platform for identifying the uTISs, and could be partially recapitulated in different biological contexts.

Furthermore, we reasoned that ribosome profiling [59] experiments, which provide a way to measure translational efficiency based on RNA-seq of Ribosome-Protected mRNA Fragments (RPFs), could provide an independent evidence of the presence of potential enrichment of alternative translation initiation in the mRNAs of our interest. With this aim, we downloaded Reads Per Kilobase per Million mapped reads (RPKM) data corresponding to the 5’ UTRs of the mRNAs modelled in the normal tissue, NCI-60 or CPTAC CRC panels from thirteen ribosomal profiling studies conducted in human normal cell lines from RPFdb, a resource hosting data based on deep sequencing of ribosome protected mRNA fragments [60]. For each panel and ribosomal profiling study, we then checked the correlation between improvement in predictive accuracy and ribosomal coverage (RPKM values) of the 5’ UTRs of modelled mRNAs, which was statistically significant only for the normal tissue panel (Spearman’s correlation coefficient, p < 0.05, **Table B in S1 Text**). Results from this final assay reflected the different extents to which the RBP^plus^ models were found to improve accuracy in predicted protein abundance in the three panels, with the normal tissue panel showing better RBP^plus^ model performances.

### Prioritization of associated RBPs

**Fig 5:**
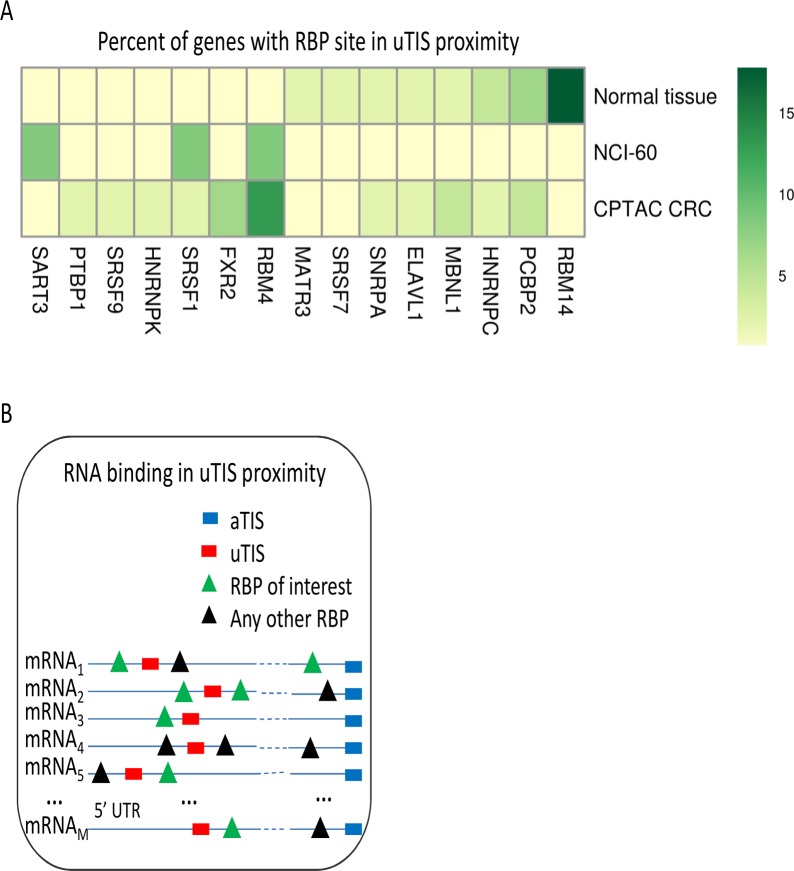
Prioritization of RNA binding proteins. Candidate RBPs are identified analysing the binding sites of each RBP in the RBP binding sites situated nearest to the uTISs of the mRNAs where the RBP^plus^ model improves accuracy pf predicted protein abundance. **(A)** The heat map displays the percentages of genes where each RBP showed the minimal distance between a RBP binding site and an uTIS. **(B)** The inset displays the criterion of minimal distance between RBP binding sites and uTISs used to identify RBPs. RBPs are shown if they resulted to recognize the binding sites closest to the uTISs of mRNAs in at least one of the three panels.

Our results indicate that the presence of uTISs is a common feature of those mRNAs where RBPs included in the RBP^plus^ model improved predictive accuracy compared to the RNA^only^ model. Even if this association does not mean a biological link between RBPs and uTISs, it suggests that translational regulation of the main ORF could be exerted by some of the considered RBPs through an uTISs. A potential, direct mechanism for this regulation could be steric control of uTIS elements by local RBP binding. We adopted this hypothesis to attempt an initial prioritization of RBPs. In case of steric control, RBP binding sites need to be in the proximity of a uTIS. No demonstrated example of such a control is present, at the best of our knowledge, in the literature. A functional proximity between uTISs and RBP binding sites has been reported only in one study involving the Drosophila SXL protein, but in this case the uTIS defines a uORF [61]. We selected the closest RBP binding site to each uTIS identified in a gene where the RBPs in the RBP^plus^ model improved the accuracy in predicted protein abundance relative to the RNA^only^ model (p < 0.05 by randomization of proteins). We then ordered the RBPs according to the proportion of genes where they were inferred to recognize the binding sites located nearest to the uTISs. This analysis led us to prioritize the 15 RBPs inferred to bind the identified mRNAs (**Fig 5**). Of them, PCBP2 has been previously implicated in translational control by an internal ribosomal entry site (IRES) [62].

## Discussion

Although transcriptomic and proteomic assays are rarely integrated in large-scale studies, such integration provides a still unexploited instrument to study post-transcriptional control in a large-scale perspective. We performed an integrative analysis of matched RNAseq-based transcript and MS-based protein profiles to assess potential interaction between RBPs and mRNAs to determine protein abundance, beyond the contribution of transcript abundance. The pool of adequately measured proteins, as expected, was a fraction of the transcriptome coverage and was functionally biased for certain GO themes. RNA^only^ and RBP^plus^ model were fitted for each mRNA/protein pair employing linear regression. To define the extent to which the RBP^plus^ model improves the accuracy in predicted protein abundance over the RNA^only^ model, we harmonized our regression approaches for the RNA^only^ and RBP^plus^ models, so that if RBPs are useless covariates, the RBP^plus^ model is expected to converge to RNA^only^ one. We carefully checked the extent to which the effect produced by the RBPs, which were inferred to bind the modelled mRNAs, can be recapitulated by randomly sampled predictors, assessing statistical significance of improvement in predictive accuracy by empirical randomization tests. Our analysis suggested a large room for improvement over the RNA^only^ models, but the improvement in accuracy of predicted protein abundance achieved by the RBPs included in the RBP^plus^ models could be reconstructed by randomly sampled proteins in the largest majority of the genes that we could model. Indeed, gene-level randomization tests identified a small fraction of genes where the impact of inferred RBP-mRNA interaction on improved predictive accuracy was statistically significant. Measuring the association of the improvement in accuracy of predicted protein abundance with mRNA features led to identify uTISs as a common feature of the genes where RBPs were shown to be informative.

Recently, allele-specific translational efficiency in an F1 hybrid mouse was determined by transcriptome and polysome profiling, and an analysis of sequence features of mouse genes with biased allelic translation revealed that out-of-frame uTISs could affect translational efficiency [63]. The impact of RBPs on the improvement in accuracy of predicted protein abundance was limited to a fraction of mRNAs, and it was dependent on the number of uTISs present in mRNA 5’ UTRs but not on the strength of the downstream aTISs. Our analysis cannot provide for a potential mechanism or decide for a direct versus an indirect effect, but given these features one of the possibilities is that some of the informative RBPs could modulate translation initiation of the downstream ORFs by simply either repressing or promoting alternative, uTIS-based, translation initiation.

Regulation of translation initiation in mammalian cells by interaction of RBPs with mRNA 5’ UTRs has been rarely documented, with a few examples involving the interaction between RBPs and internal ribosomal entry sites (IRESs) of specific stress-related mRNAs (reviewed in [64]), or the interaction between the IRP-1 RBP and the iron-responsive element (IRE) of the ferritin mRNA [35]. But no uTIS-dependent effect has been found in these well-studied cases. The presence of uORFs is known to regulate translation of primary downstream ORFs by operating via decay, re-initiation, or peptide-mediated ribosomal stalling during uORF translation [55],[65],[25]. Although uORFs can regulate protein levels without involving RBPs [65], an already cited previous study in Drosophila offers an example where the SXL RBP promotes translation initiation at the uORF of the msl-2 and Irr47 transcripts [61], which thus results in translational repression. More recently, the DENR-MCT-1 complex has been identified as a regulator of eukaryotic uORF-dependent translation re-initiation of a specific group of mRNAs [34]. But to our knowledge no RBP-induced, non uORF-mediated translational control mechanism in uTIS-endowed loci has yet been identified.

Based on the hypothesis of a direct mechanism of RBP control of uTISs, such mechanism could be sensitive to changes in the position and spacing between RBP binding sites within the mRNA 5’ UTR. We therefore used the criterion of spatial proximity to prioritize the RBPs which were shown to help in predicting protein abundance. Of course, we cannot exclude that this control could be due to 3’ UTR binding, considering also that 3’ UTR-acted and RBP-mediated translational initiation controls are an established model. Yet, this model has been proposed [25] on the basis of few notable cases in Drosophila [66],[67],[68],[69] and Xenopus [70] translational control always during development and differentiation. In these examples, the RBP ensures specificity to the regulation of translation by binding sites within the 3’ UTR of the mRNA and contributes to the formation of a closed loop which precludes formation of the initiation complex eIF4F, therefore exerting an inhibitory effect on translation. It is worth considering that, again, in this “classical” model no role is attributed to uTISs.

With the limitations highlighted in mind, the study presented here allowed us to estimate the impact of RBP-mRNA interactions on quantitative relationships between mRNA and protein abundances. RBPs were shown to help in predicting protein abundance relative to an RNA^only^ model, but not relative to randomly selected proteins, in the majority of considered mRNAs. Nonetheless, our analysis identified genes for which inferred RBP-mRNA interactions were informative. The association between the improvement in accuracy of predicted protein abundance and uTISs suggests that RBPs could modulate the expression of these genes by mediating alternative translation regulation. The usefulness of RBP^plus^ models need to be further tested as soon as suitable datasets are produced by RNAseq and MS-based technologies. The pervasive presence of conserved uTISs in the human transcriptome, which has been recently revealed by ribosome profiling and related approaches [52],[62],[63], awaits a clarification of their functional role.

## Materials and Methods

### Transcriptome and proteome datasets

Matched transcriptome and proteome profiles were downloaded in the processed form provided by three independent datasets: 1) a panel of twelve human normal tissues [43], 2) the 59 samples from the US National Cancer Institute (NCI)-60 dataset [44], and 3) 87 colorectal cancer (CRC) samples profiled by The Cancer Genome Atlas (TCGA) in combination with the Clinical Proteomic Tumour Analysis Consortium (CPTAC) [45].

Processed data derived from gene expression analysis in the normal tissue panel were downloaded from the online Supplementary Information of the study [43]. Normalized transcriptome data for NCI-60 cell lines were obtained from the Gene Expression Omnibus (series accession number GSE32474), while processed proteome data were downloaded from http://wzw.tum.de/proteomics/nci60. Processed proteome data for TCGA colorectal cancer samples were downloaded from the online Supplementary Information of the study [45], while processed transcriptome data were downloaded from TCGA (http://cancergenome.nih.gov/).

In the normal tissue panel and CRC panel, transcript abundance data were obtained by RNA sequencing (RNA-seq) and expressed as Fragments Per Kilobase per Million, log-base-10 FPKM. NCI-60 transcriptome profiles were obtained by microarray. Normal tissue proteome profiles were obtained by the intensity-based Absolute protein Quantification method, and expressed as log-base-10 iBAQ. NCI-60 and CPTAC CRC proteome profiles were based on liquid chromatography-tandem mass spectrometry (LC-MS/MS)-based shotgun proteomic analysis. Intensity- and spectral count-based label-free quantifications were used to obtain protein abundance in the NCI-60 cell lines and in the CPTAC CRC specimens, respectively.

In the normal tissue panel we excluded genes and proteins below the detection limit in more than three out of twelve tissues at either transcriptome or proteome level; in the NCI-60 and CRC panels we excluded genes below the detection limit in more than five out the total number of specimens at either the transcriptome or proteome level. Genes below the detection limit were assigned zero values or Not Available (NA) labels in the files processed data were acquired from.

Within each panel, we applied inter-sample normalization by mRNA and protein mean-centring per sample.

### Inference of the interaction between RNA binding proteins and mRNAs

We scanned non-redundant 5’ and 3’ untranslated region (UTR) sequences of the genes profiled at both the transcript and protein levels with positional weight matrices (PWMs), which represent RNA sequence binding specificities of RBPs derived from RNAcompete [31] data and which are available through the cisBP-RNA database (http://cisbp-rna.ccbr.utoronto.ca/). In each panel of matched transcriptome/proteome datasets, the inference of RBP binding sites in mRNA UTRs was restricted to the subset of RBPs which were detected both at the transcript and at the protein level. For each considered RBP, RBP binding sites as well as corresponding q-values were obtained using the FIMO algorithm [71] of the MEME toolkit (http://meme-suite.org/) and retained at the false discovery rate of 20%.

### Model building

We built two models for each considered gene: a basic (RNA^only^) model, where the abundance of protein j in sample i (PROT_ij_) was predicted by the corresponding mRNA level only in a simple linear regression model:

$$RNA^{only}:\;PROT_{ij}=\beta_{0j}+\beta_{mRNA,j}\;mRNA_{ij}+\varepsilon_{ij};\;\varepsilon_{ij}{∼}^{iid}\;N(0,\;\sigma_{i})$$

where β_0j_ is the intercept term, β_mRNA,j_ is the regression coefficient for the mRNA predictor and the error term ε_ij_ is an independent and identically distributed (iid) random variable following a normal distribution of mean 0 and standard deviation σ. This model was fit for each mRNA/protein pair. An RBP-inclusive (RBP^plus^) multiple linear regression model was also fitted for each mRNA/protein pair:

$$RBP^{plus}:\;PROT_{ij}=\beta_{0j}+\beta_{mRNA,j}\;mRNA_{ij}+\beta_{RBP,jk}\;RBP_{ijk}+\varepsilon_{ij};\;\varepsilon_{ij}{∼}^{iid}\;N(0,\;\sigma_{i})$$

where β_RBP,jk_ is the regression coefficient for the k^th^ RBP of mRNA j. This model was fitted by maximum penalized likelihood with Ridge or LASSO penalty applied to RBPs but not to mRNA measurements, using the pensim R package [72], which acts as a wrapper providing nested cross-validation to the penalized R package [73]. In the nested cross-validation scheme, test samples are held out for accuracy estimation in the outer layer of cross-validation, and penalty parameters are tuned in the inner layer of cross-validation within training samples only. In the outer layer of cross-validation, we used 5-fold for the three panels.

By not penalizing mRNA measurements, the model can be expected to converge to the RNA^only^ model in the absence of informative RBP protein measurements. Both Ridge and LASSO penalty help control of over-fitting of high-dimensional data; LASSO additionally provides feature selection by setting the coefficients of most covariates to exactly zero.

We fitted these two models, independently for each gene inferred to be bound by an RBP and in each tissue/cell panel where both transcript and corresponding protein met the missingness requirements described above.

The change in accuracy of predicted protein abundance obtained by the RBP^plus^ model relative to the RNA^only^ model of each considered gene was quantified by the difference in the R^2^ coefficients between the RBP^plus^ and RNA^only^ models. This analysis used the following R^2^ coefficient definition:

$$R^{2}=1-\frac{\sum_{i}{\left(y_{i}-f_{i}\right)}^{2}}{\sum_{i}{\left(y_{i}-\langle y\rangle \right)}^{2}}$$

where y_i_ the i-th observation, <y> is the mean of the observations, and f_i_ is the i-th prediction.

We evaluated the statistical significance of the improvement in accuracy of predicted protein abundance attained by the RBP^plus^ model relative to the RNA^only^ model across the genes considered in each separate panel by Wilcoxon signed-rank test.

### Model assessment by randomization of tissues and RNA binding proteins

Empirical randomization tests were used to determine whether the accuracy in predicted protein abundance achieved by the RBP^plus^ model of an individual gene was statistically significantly better relative than that expected for randomized RBP^plus^ models of the gene. For each considered gene, we obtained 1000 randomized versions of the RBP^plus^ model by 1) randomly sampling a number of protein predictors equal to the number of actual RBPs inferred to bind the mRNA UTRs, and 2) by permuting the protein levels of inferred RBPs across samples. As the actual RBP^plus^ models, each randomized RBP^plus^ model (by sample permutation or randomly sampling of protein predictors) was fitted by maximum penalized likelihood with Ridge penalty applied to RBPs but not to mRNA measurements in nested cross-validation scheme. **Fig 3**illustrates the two randomization schemes.

The p-value of the R^2^ value observed for the actual RBP^plus^ model of each considered gene was defined by the probability of sampling a R^2^ value from the null distribution of R^2^ values that is higher than the observed R^2^. The RBP^plus^ model of a gene was deemed to improve the accuracy in prediction of protein abundance if the RBP^plus^ model accuracy was higher than that of the RNA^only^ model and if the probability of attaining accuracy higher than that of the RBP^plus^ model by randomly sampling protein predictors was < 0.05. Since our analysis involved multiple hypotheses testing, we reported false discovery rate by the Storey’s q-value method implemented in the *qvalue* R package [50].

### Model assessment by cross-panel validation

We studied cross-panel model transferability of models trained using only the RBPs profiled in both of each pair of panels. For each considered mRNA, we developed the RNA^only^ and RBP^plus^ models using all samples in a training panel, and tested them using all samples in the testing panel. The procedure was repeated for all possible combinations of training and test panels. We estimated model transferability computing Spearman’s correlation coefficient of protein predictive accuracies between the RBP^plus^ models trained in a chosen panel and the RBP^plus^ models trained in each of the other two panels.

### Gene functional enrichment/depletion analysis

Functional enrichment/depletion analysis was based on the Biological Process categories of the generic Gene Ontology (GO) slim, a cut-down version of the Gene Ontology annotations (http://geneontology.org/) and used hypergeometric test. Functional analysis was used 1) to assess over-/under-representation of GO themes in the genes which turned out to be adequately measured relative to the total of genes which were profiled at the mRNA/protein levels, and 2) to assess over-/under-representation of GO themes in the genes where RBP^plus^ models were found to be informative relative to the total of modelled genes.

### Analysis of correlation between improvement in predictive accuracy and post-transcriptional gene features

We surveyed appropriate data sources to gather several gene annotations relevant to post-transcriptional regulation of gene expression in mammalian cells (**Table 1**). We quantified the selected features in the mRNAs modelled in the normal tissue panel as follows. Normalized lengths of the coding sequence as well as of the 5’ and 3’ UTRs were calculated for each mRNA according to the sequence annotations (hg38 assembly) available at the UCSC Genome Browser (https://genome.ucsc.edu/). Local folding energy was computed within a window of 30 nucleotides upstream and downstream of the annotated translation initiation site of the modelled mRNAs using the RNAfold algorithm of the Vienna RNA package (www.tbi.univie.ac.at/RNA/). Transcript half-life measures were acquired by two distinct studies which relied, respectively, on biosynthetic labelling of newly transcribed RNA and estimation of newly/total RNA ratio in human B cells [74], and on transcription blocking in HepG2 and Bud8 cell lines [75]. A measure of efficiency of start codon recognition of primary ORFs was derived from a quantitative analysis of translation initiation sites by FACS-seq, Fluorescence-Activated Cell Sorting and high-throughput DNA sequencing [52]. The tRNA adaptation index (tAI), an estimate of the translational optimality of a coding sequence to cellular tRNA pools was computed by the codonR software [76]. Annotation of upstream translation initiation sites (uTISs) was derived by Global Translation Initiation sequencing (GTI-seq) in HEK293 cells and downloaded from the TISdb database [77]. We included an additional mapping of upstream translation initiation sites which was obtained by Quantitative Translation Initiation sequencing (QTI-seq) in HEK293 cells [58].

Upstream Open Reading Frames (uORFs) were defined by: (i) an uTIS out-of-frame at the 5’ UTR, with a stop-codon, in the same frame, downstream of it, and with a minimal length of nine nucleotides, (ii) an uTIS in-frame at the 5’UTR with a stop codon in frame after the main stop codon or before the main start codon.

We used Spearman’s correlation coefficient to estimate the correlation of the change in accuracy of predicted protein abundance with each aforementioned feature. Furthermore, we used Fisher’s test to assess the enrichment of the genes where the RBP^plus^ model was found to be informative in uTIS-containing genes as well as in uORF-containing genes. Testing was performed for uTISs identified by GTI-seq and QTI-seq technologies and for each panel of paired mRNA/protein datasets.

### Analysis of association between upstream translation initiation and RNA binding proteins

RBPs were prioritized by an analysis of the frequency at which the binding sites of an RBP occur in the proximity of uTISs of mRNAs. We identified the closest RBP binding site to each uTIS present in the 5’ UTR of each mRNA. We then quantified the frequency of the binding sites of each RBP in the binding sites situated nearest to the uTISs overall mRNAs. RBPs were ordered according to the number of genes where they were found to recognize the binding sites closest to the uTISs.

### False discovery rate control

In the contexts where multiple tests were performed, raw P-values were adjusted by the Benjamini-Hochberg method for controlling false discovery rate at 5%.

## Supporting Information

S1 Fig The fraction of genes is displayed by the number of samples where the mRNA and protein levels of the gene were not detectable. (TIF)

S2 Fig Shift in mRNA and protein levels upon gene selection. (**A**) Distributions of the median logarithmic mRNA abundances of all genes (dashed line) and of the genes selected on the basis of the detection frequency across the samples in each panel (solid line). (**B**) Distributions of the median logarithmic protein abundances of all genes and of the genes selected on the basis of the detection frequency across the samples in each panel.
(TIF)

S3 Fig Functional depletion/enrichment in Gene Ontology categories for adequately quantitated genes. Functional Gene Ontology enrichment analysis of the genes selected for modelling in each panel, showing depleted or enriched GO slim categories (p < 0.05). A Gene Ontology category is shown if false discovery rate meets threshold in at least one panel.
(TIF)

S4 Fig mRNA abundance quantification in each panel. mRNA expression data are unmodified with respect to the original publication. **(A)** Distribution of Fragments Per Kilobase per Million (FPKM) from RNA-seq experiments of all 12 normal tissue samples. **(B)** Distribution of mRNA intensity from microarray profiling experiments of all 59 NCI-60 cell lines. **(C)** Distribution of Fragments Per Kilobase per Million (FPKM) from RNA-seq experiments of all 87 CPTAC CRC samples.
(TIF)

S5 Fig Protein abundance quantification in each panel. Protein expression data are unmodified with respect to the original publication. **(A)** Distribution of protein intensity from proteome profiling experiments of all 12 normal tissue samples. **(B)** Distribution of protein intensity from proteome profiling experiments of all 59 NCI-60 cell lines. **(C)** Distribution of spectral counts from proteome profiling experiments of all 87 CPTAC CRC samples.
(TIF)

S6 Fig Inter-sample normalization effects on model performances. Distribution of R^2^ achieved by the RNA^only^ (dashed line) and RBP^plus^ (solid line) models according to different types of inter-sample normalization. Shown are p-values of Wilcoxon signed-rank tests to assess differences in the ranks of predictive accuracy between the RNA^only^ and RBP^plus^ models based on each type of inter-sample normalization.
(TIF)

S7 Fig Influential observations are sparse in all the three panels. Heat maps display Cook’s distance values for each gene and sample.
(TIF)

S8 Fig Predicted RBP-mRNA interactions are combinatorial. Distribution of number of RBPs inferred per mRNA using the thresholds of 5% or 20% to the false discovery rate on RBP binding sites.
(TIF)

S9 Fig Network clustering analysis delivers modules of RBP-RNA interactions yielding improvement in protein prediction accuracy. (**A**) Node colour distinguishes source (RBP predictor) and target (modelled gene) nodes. An edge indicates that the RBP is predicted to bind the mRNA. A target node weight is introduced to represent the improved accuracy in the protein abundance prediction of the RBP^plus^ model in comparison to the RNA^only^ one, whereas an edge weight represents the regression coefficient of the RBP in the RBP^plus^ model of the target mRNA. Only statistically significant modules totalizing mean edge weight and entropy values above median values are displayed. (**B**) Gene-wise correlations between experimental protein levels and protein levels predicted, respectively, by the RBP^plus^ and the RNA^only^ models are shown for each module. The RBP^plus^ model improves the correlation between inferred and observed protein levels in all modules. The modules where the improvement is statistically significant display pincers on the top of the corresponding pairs of boxplots.
(TIF)

S10 Fig Improvement of RBP^plus^ model relative to RNA^only^ model is independent of stringency to infer RBP-mRNA interactions. Shown are the distributions of protein predictive accuracy (R^2^) obtained by the RNA^only^ models as well as by the RBP^plus^ models using RBP-mRNA interactions inferred at different false discovery rates (FDRs). We tested differences in rank of protein predictive accuracies between RNA^only^ models and RBP^plus^ models at different FDR values by the Wilcoxon signed-rank test. P-values are shown and colour-coded in figure.
(TIF)

S11 Fig RBP^plus^ models fitted by LASSO ensure better protein predictive accuracy relative to the RNA^only^ models. The distributions of protein predictive accuracy (R^2^) for the RBP^plus^ models fitted with Ridge and LASSO penalty are shown with the R^2^ distribution for the RNA^only^ models. Wilcoxon signed-rank test was used to test differences in rank of the protein predictive accuracy for the RNA^only^ models and the RBP^plus^ models, which were fitted by either penalty. Test’s P-values are colour-coded according to the penalty used to fit RBP^plus^ models.
(TIF)

S12 Fig **(A)** RBP^plus^ models fitted with Ridge or LASSO penalty ensure comparable protein predictive accuracies. Shown are the distributions of R^2^ obtained by the RBP^plus^ models fitted with Ridge or LASSO penalty. Wilcoxon signed-rank test was used to test differences in rank of the protein predictive accuracy for the RBP^plus^ models fitted by Ridge or LASSO penalty. Test’s P-values are shown. **(B)** Distribution of the fraction (%) of predictors selected by the RBP^plus^ models fitted with LASSO penalty with respect to the predictors used in the RBP^plus^ models fitted with Ridge penalty.
(TIF)

S13 Fig Gene Ontology categories in the Biological Process domain overrepresented (p < 0.05) in genes where the RBP^plus^ model achieved better protein predictive accuracy than expected for RBP^plus^ models randomized by randomly sampling protein predictors. (TIF)

S14 Fig Protein predictive accuracy (R^2^) and number of RBPs in the RBP^plus^ models do not correlate. Correlation is estimated by Kendall’s tau coefficient in all three panels.
(TIF)

S15 Fig Overlap of modelled genes across panels. Jaccard index of modelled genes between each pair of panels included in our analysis.
(TIF)

S16 Fig Cross-panel transferability of models. RBP^plus^ models show some transferability across tissue panels. Better transferability is observed from NCI-60 and CPTAC CRC panels to normal tissue panel. All the possible combinations of training and test panels are grouped by test panel. Shown is the Spearman’s correlation coefficient between R^2^ of RBP^plus^ models trained in the testing panel (shown in vertical axis label) and R^2^ of RBP^plus^ models trained in the remaining two panels (shown in horizontal axis labels).
(TIF)

S1 File Informative RBP^plus^ models. The table shows accuracy in predicted protein abundance achieved by the RNA^only^ and RBP^plus^ models as well as the p-value by randomization of protein predictors in the RBP^plus^ model.
(XLSX)

S1 Text Supplemental methods and results. (DOCX)
