# Supplementary figures and images for: Control of Gene Expression by RNA Binding Protein Action on Alternative Translation Initiation Sites
Source: PLoS Comput Biol. 2016 Dec 6;12(12):e1005198. doi: 10.1371/journal.pcbi.1005198 (PMC5140048; doi:10.1371/journal.pcbi.1005198)

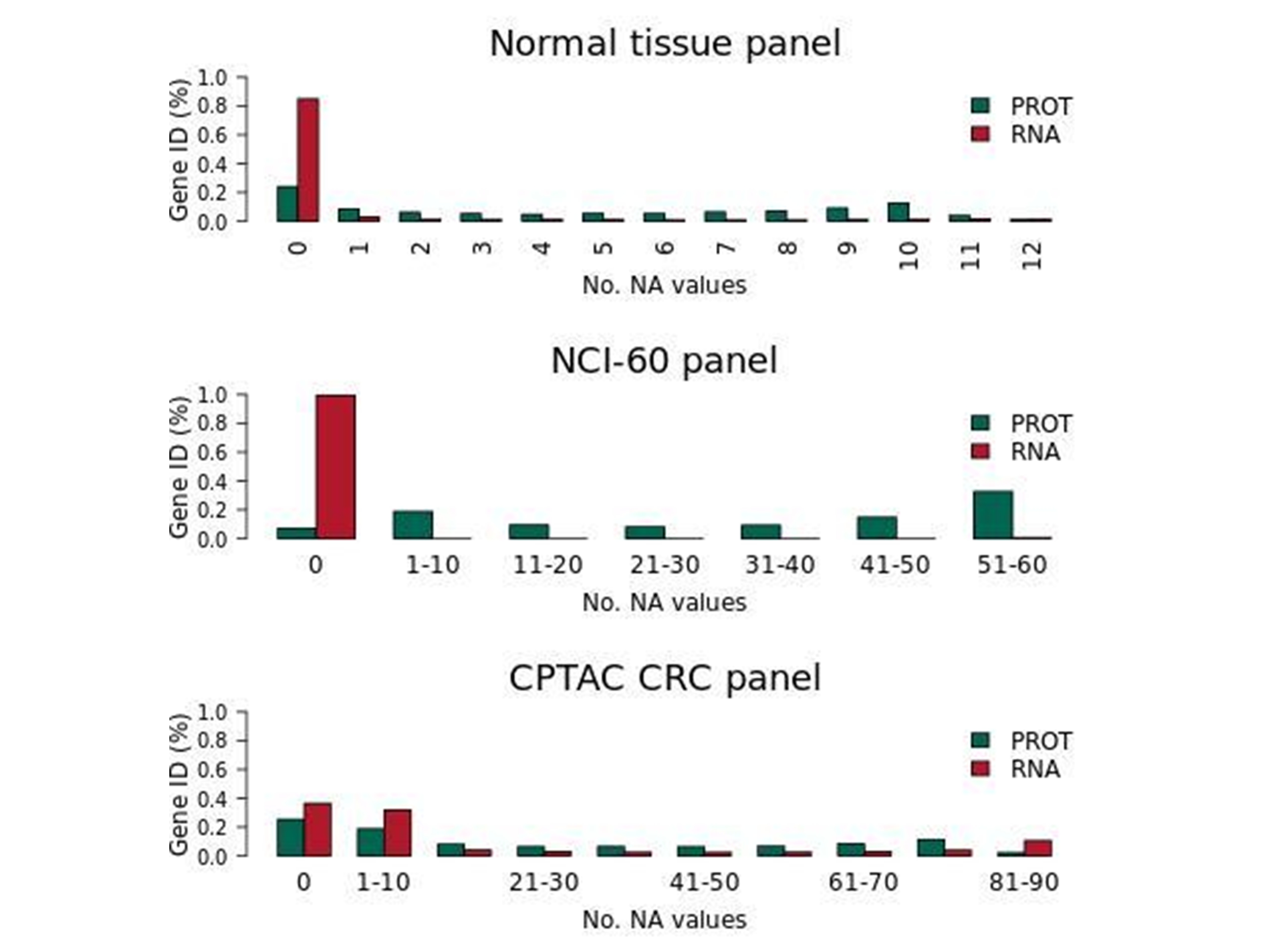

Supplement: S1 Fig — (TIF) [file pcbi.1005198.s001.TIF]

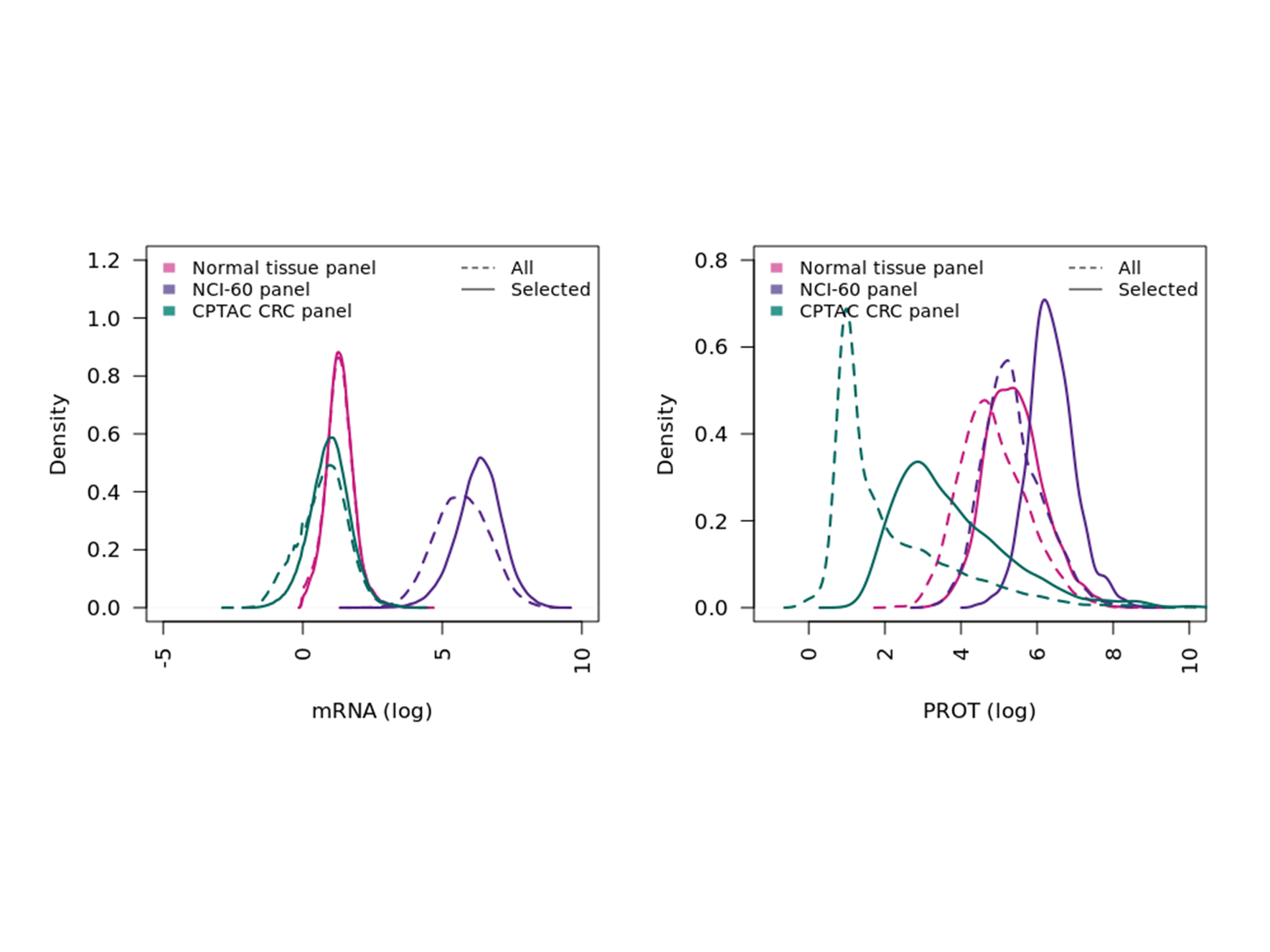

Supplement: S2 Fig — (A) Distributions of the median logarithmic mRNA abundances of all genes (dashed line) and of the genes selected on the basis of the detection frequency across the samples in each panel (solid line). (B) Distributions of the median logarithmic protein abundances of all genes and of the genes selected on the basis of the detection frequency across the samples in each panel. (TIF) [file pcbi.1005198.s002.TIF]

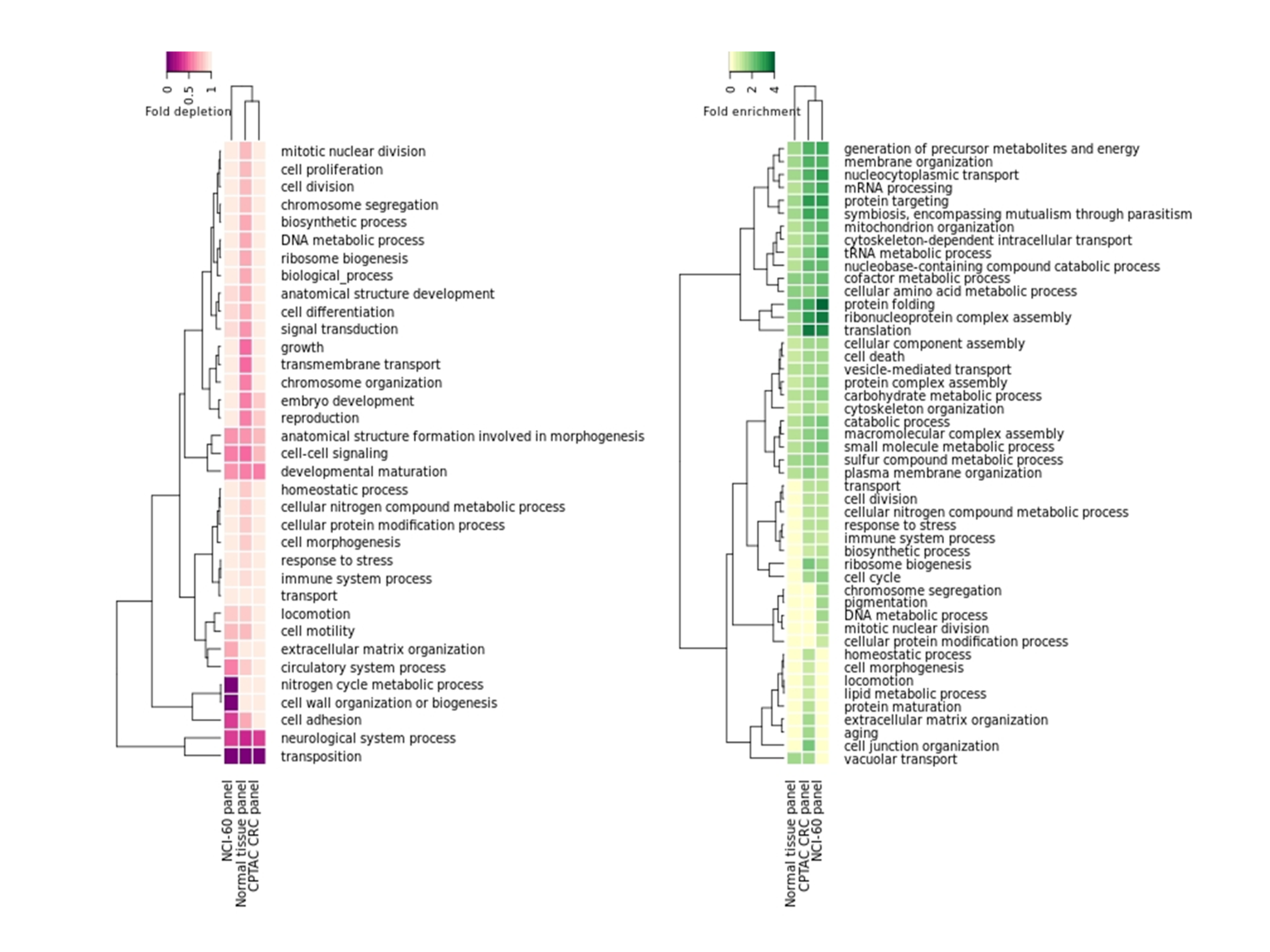

Supplement: S3 Fig — Functional Gene Ontology enrichment analysis of the genes selected for modelling in each panel, showing depleted or enriched GO slim categories (p < 0.05). A Gene Ontology category is shown if false discovery rate meets threshold in at least one panel. (TIF) [file pcbi.1005198.s003.TIF]

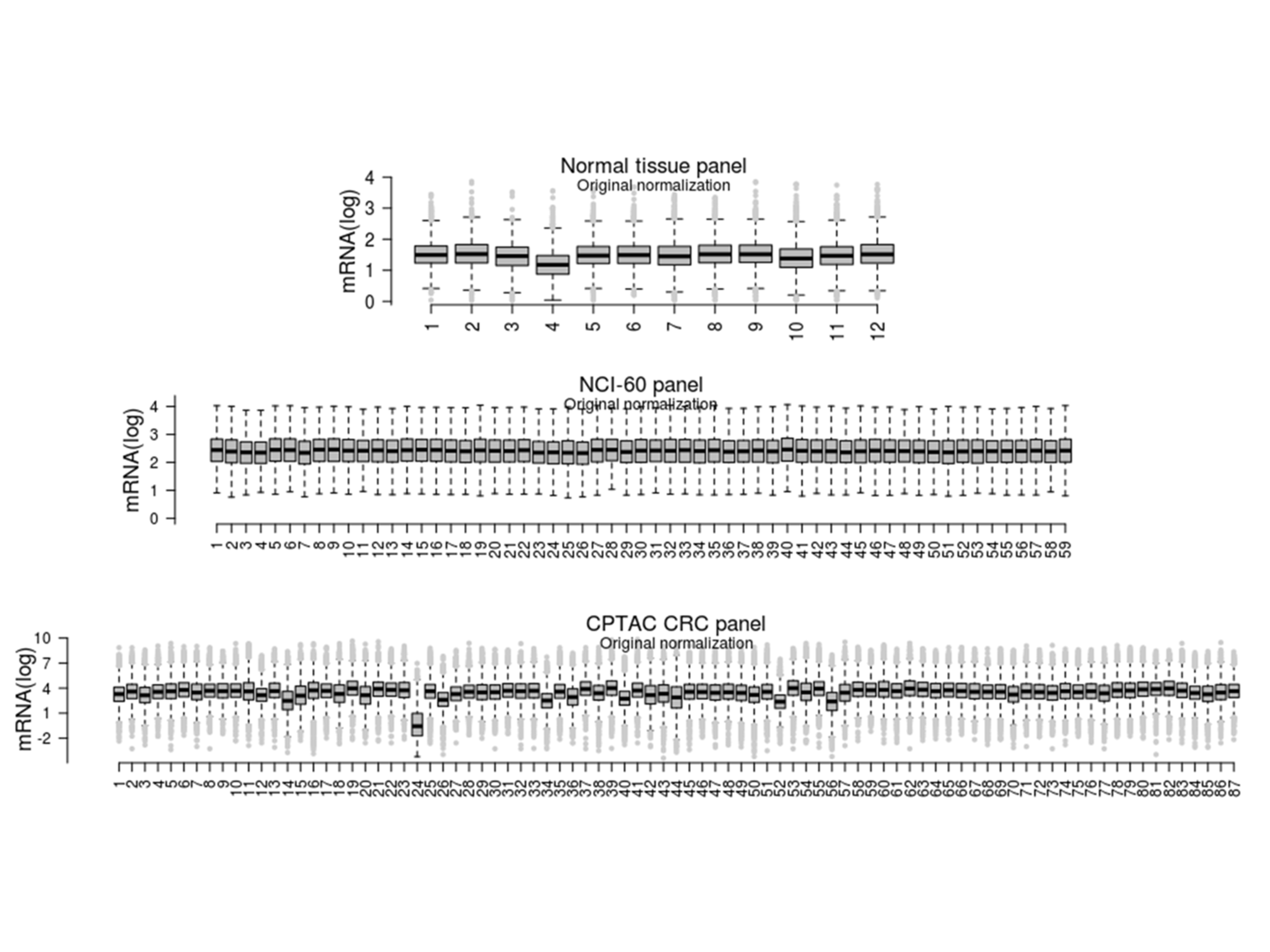

Supplement: S4 Fig — mRNA expression data are unmodified with respect to the original publication. (A) Distribution of Fragments Per Kilobase per Million (FPKM) from RNA-seq experiments of all 12 normal tissue samples. (B) Distribution of mRNA intensity from microarray profiling experiments of all 59 NCI-60 cell lines. (C) Distribution of Fragments Per Kilobase per Million (FPKM) from RNA-seq experiments of all 87 CPTAC CRC samples. (TIF) [file pcbi.1005198.s004.TIF]

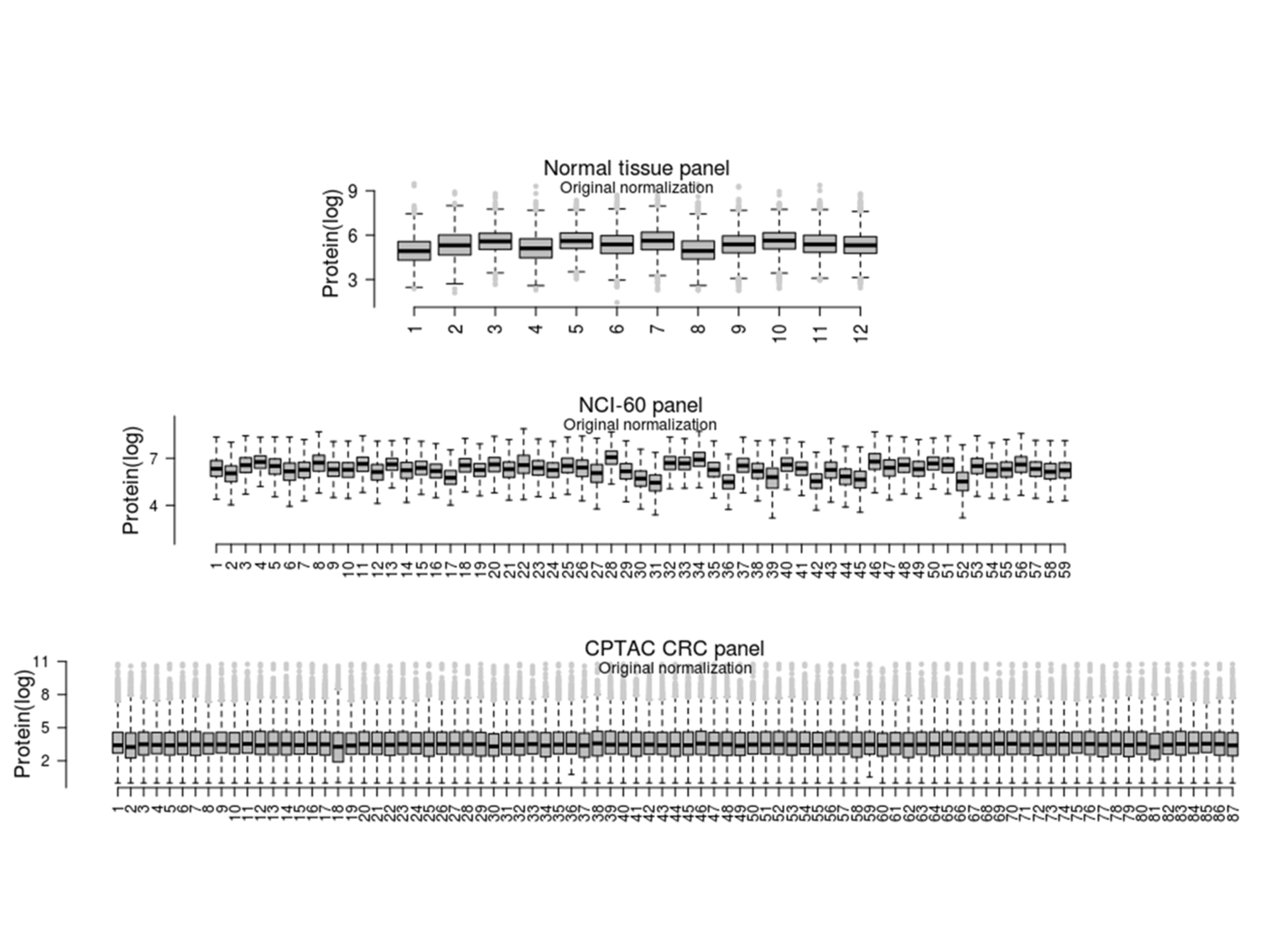

Supplement: S5 Fig — Protein expression data are unmodified with respect to the original publication. (A) Distribution of protein intensity from proteome profiling experiments of all 12 normal tissue samples. (B) Distribution of protein intensity from proteome profiling experiments of all 59 NCI-60 cell lines. (C) Distribution of spectral counts from proteome profiling experiments of all 87 CPTAC CRC samples. (TIF) [file pcbi.1005198.s005.TIF]

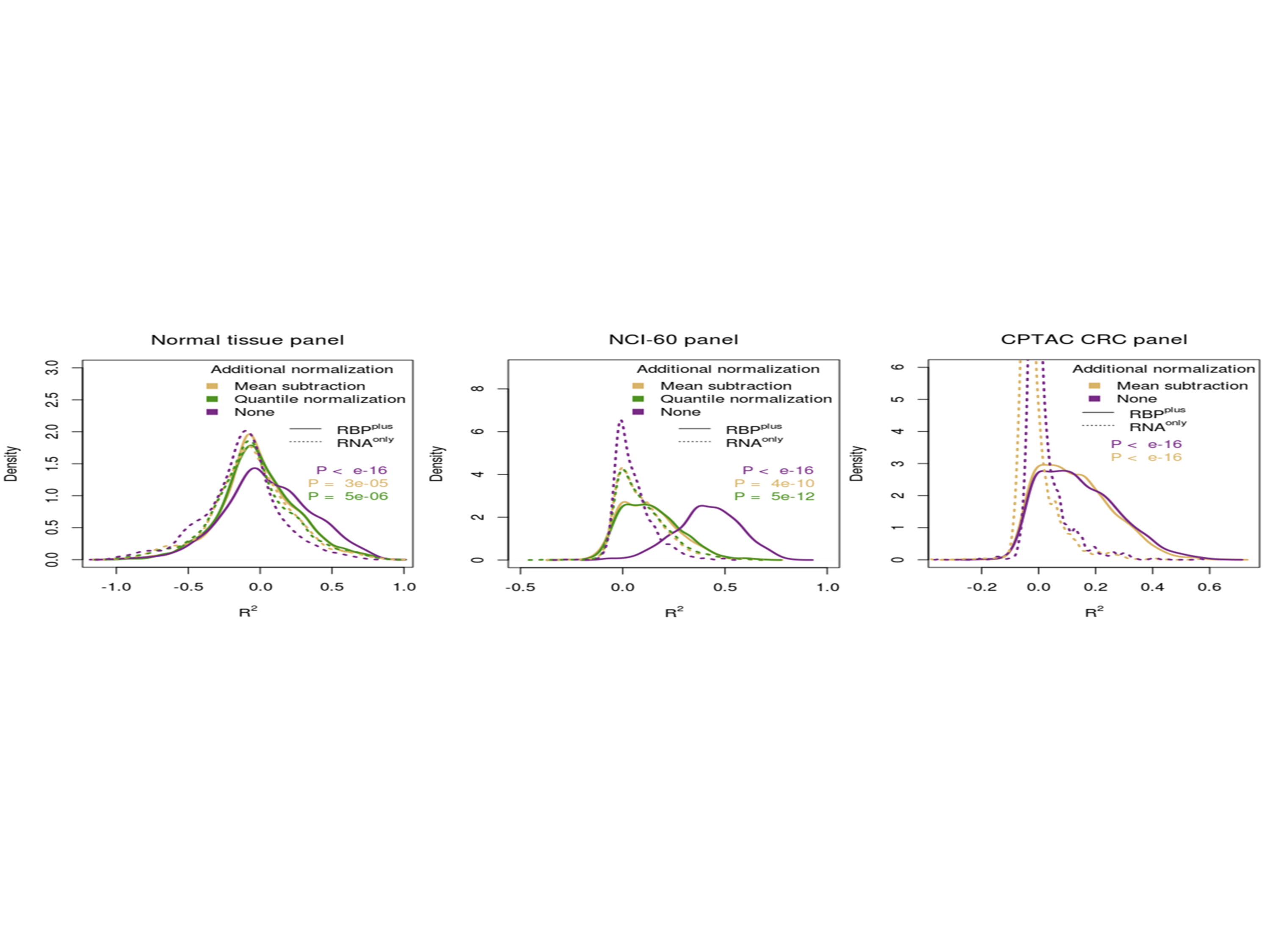

Supplement: S6 Fig — Distribution of R2 achieved by the RNAonly (dashed line) and RBPplus (solid line) models according to different types of inter-sample normalization. Shown are p-values of Wilcoxon signed-rank tests to assess differences in the ranks of predictive accuracy between the RNAonly and RBPplus models based on each type of inter-sample normalization. (TIF) [file pcbi.1005198.s006.TIF]

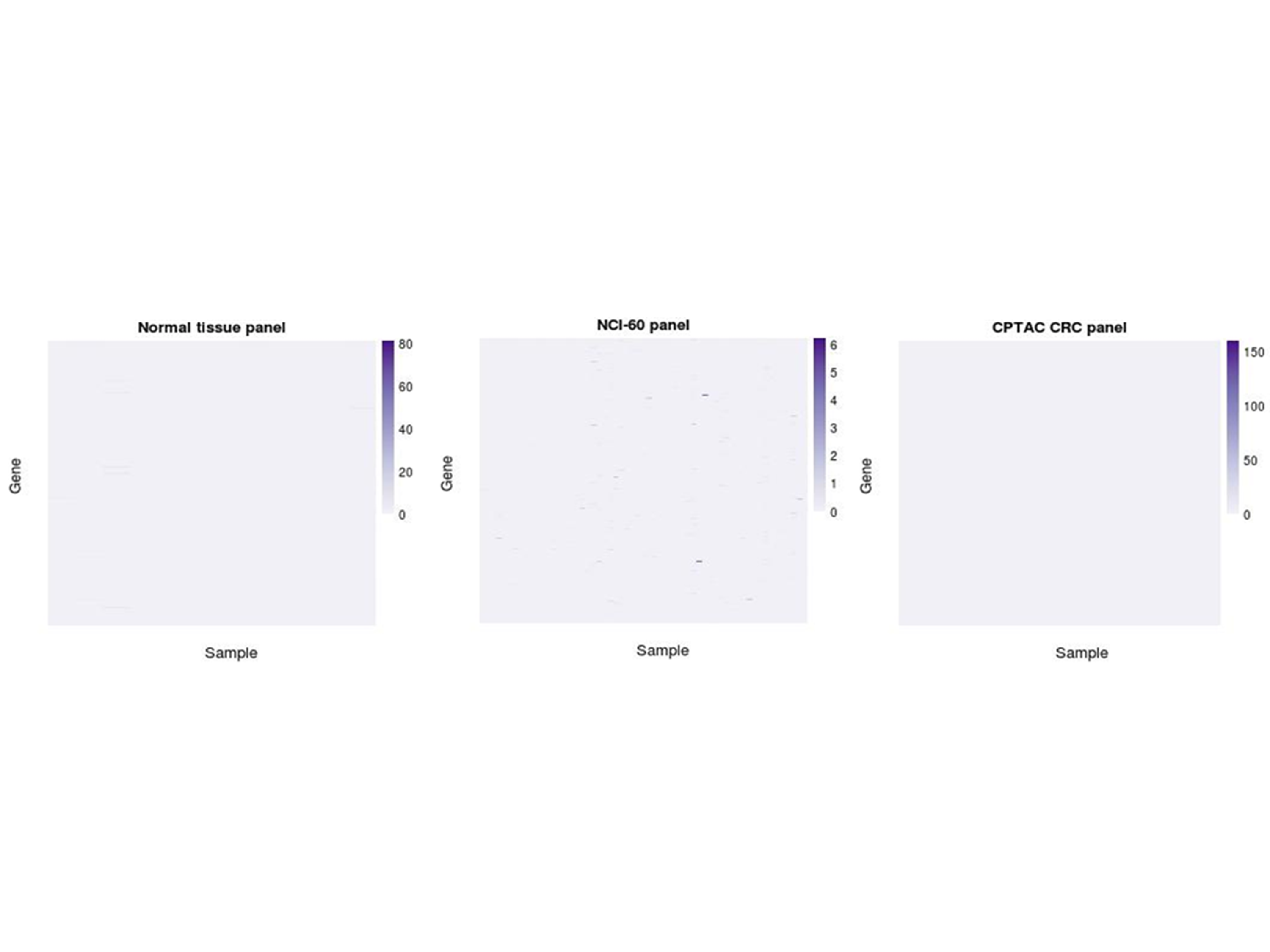

Supplement: S7 Fig — Heat maps display Cook’s distance values for each gene and sample. (TIF) [file pcbi.1005198.s007.TIF]

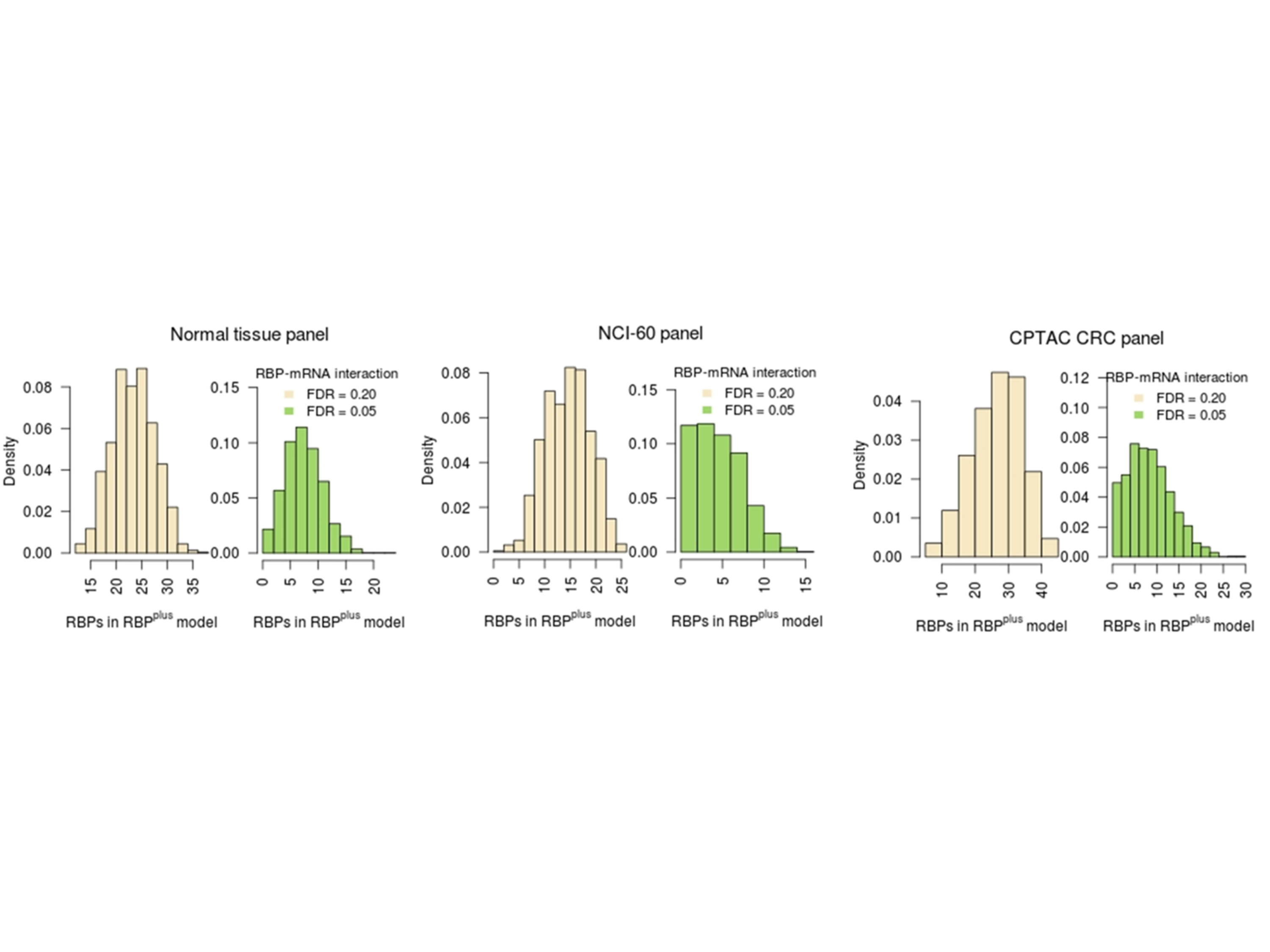

Supplement: S8 Fig — Distribution of number of RBPs inferred per mRNA using the thresholds of 5% or 20% to the false discovery rate on RBP binding sites. (TIF) [file pcbi.1005198.s008.TIF]

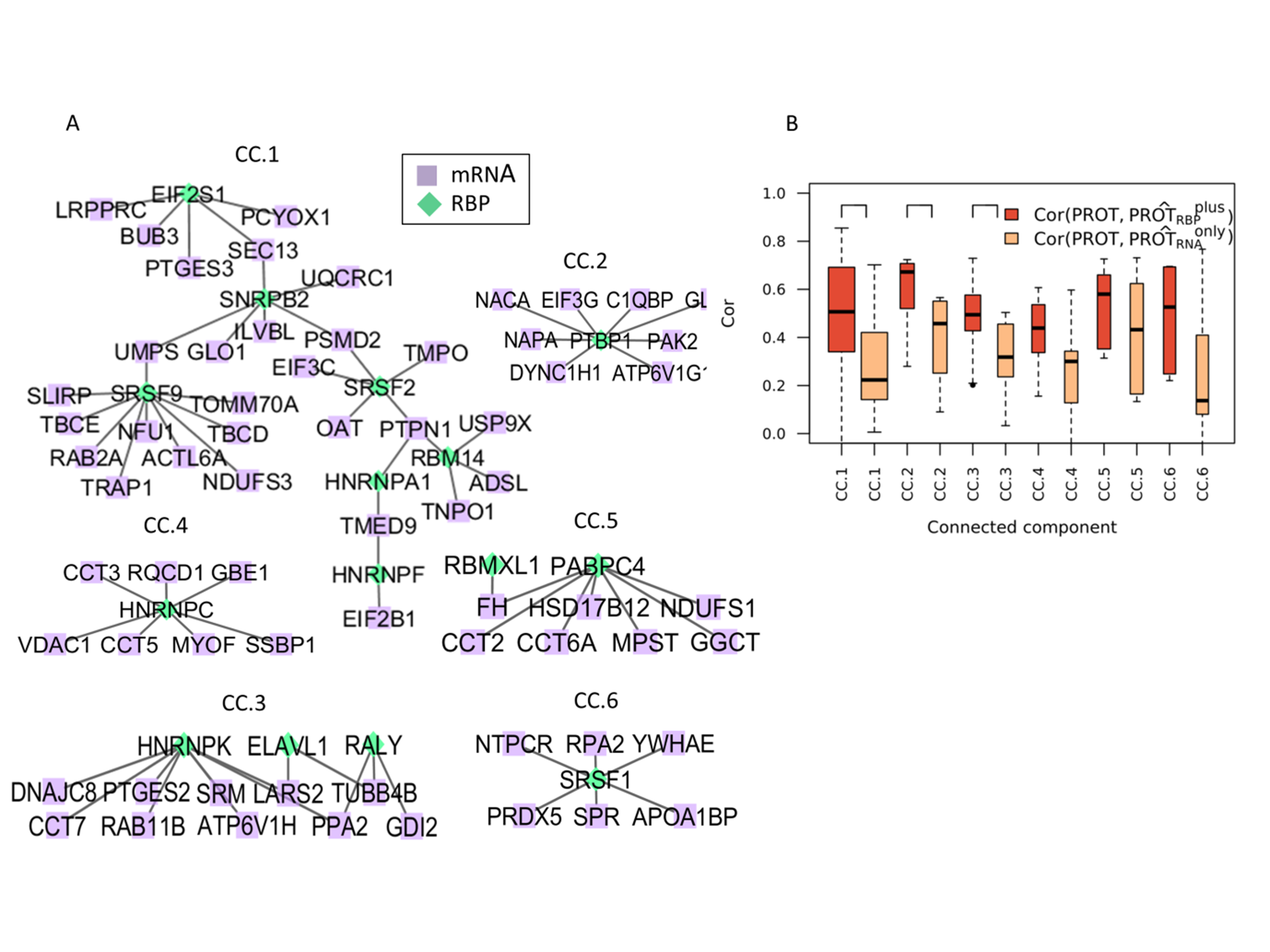

Supplement: S9 Fig — (A) Node colour distinguishes source (RBP predictor) and target (modelled gene) nodes. An edge indicates that the RBP is predicted to bind the mRNA. A target node weight is introduced to represent the improved accuracy in the protein abundance prediction of the RBPplus model in comparison to the RNAonly one, whereas an edge weight represents the regression coefficient of the RBP in the RBPplus model of the target mRNA. Only statistically significant modules totalizing mean edge weight and entropy values above median values are displayed. (B) Gene-wise correlations between experimental protein levels and protein levels predicted, respectively, by the RBPplus and the RNAonly models are shown for each module. The RBPplus model improves the correlation between inferred and observed protein levels in all modules. The modules where the improvement is statistically significant display pincers on the top of the corresponding pairs of boxplots. (TIF) [file pcbi.1005198.s009.TIF]

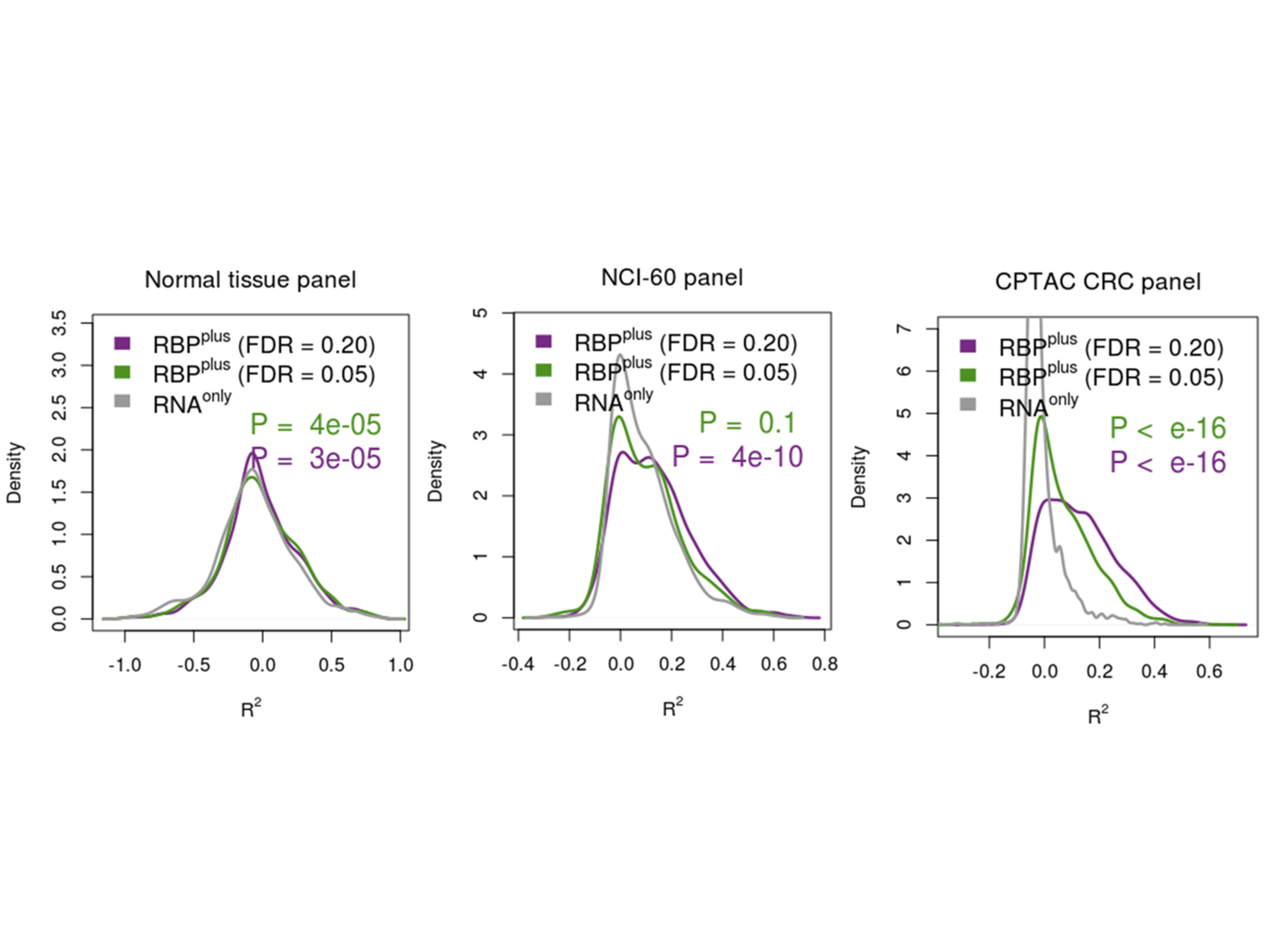

Supplement: S10 Fig — Shown are the distributions of protein predictive accuracy (R2) obtained by the RNAonly models as well as by the RBPplus models using RBP-mRNA interactions inferred at different false discovery rates (FDRs). We tested differences in rank of protein predictive accuracies between RNAonly models and RBPplus models at different FDR values by the Wilcoxon signed-rank test. P-values are shown and colour-coded in figure. (TIF) [file pcbi.1005198.s010.TIF]

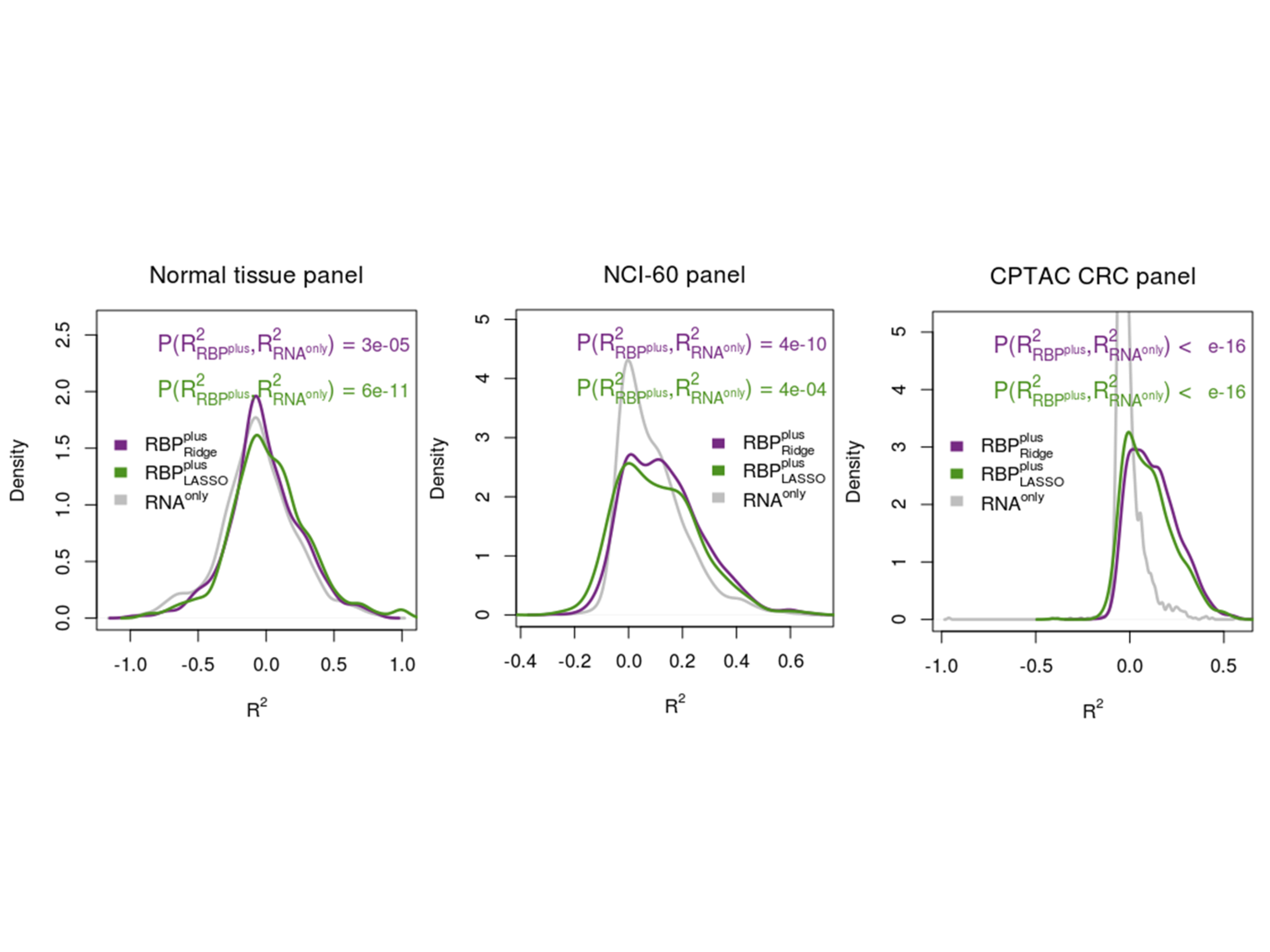

Supplement: S11 Fig — The distributions of protein predictive accuracy (R2) for the RBPplus models fitted with Ridge and LASSO penalty are shown with the R2 distribution for the RNAonly models. Wilcoxon signed-rank test was used to test differences in rank of the protein predictive accuracy for the RNAonly models and the RBPplus models, which were fitted by either penalty. Test’s P-values are colour-coded according to the penalty used to fit RBPplus models. (TIF) [file pcbi.1005198.s011.TIF]

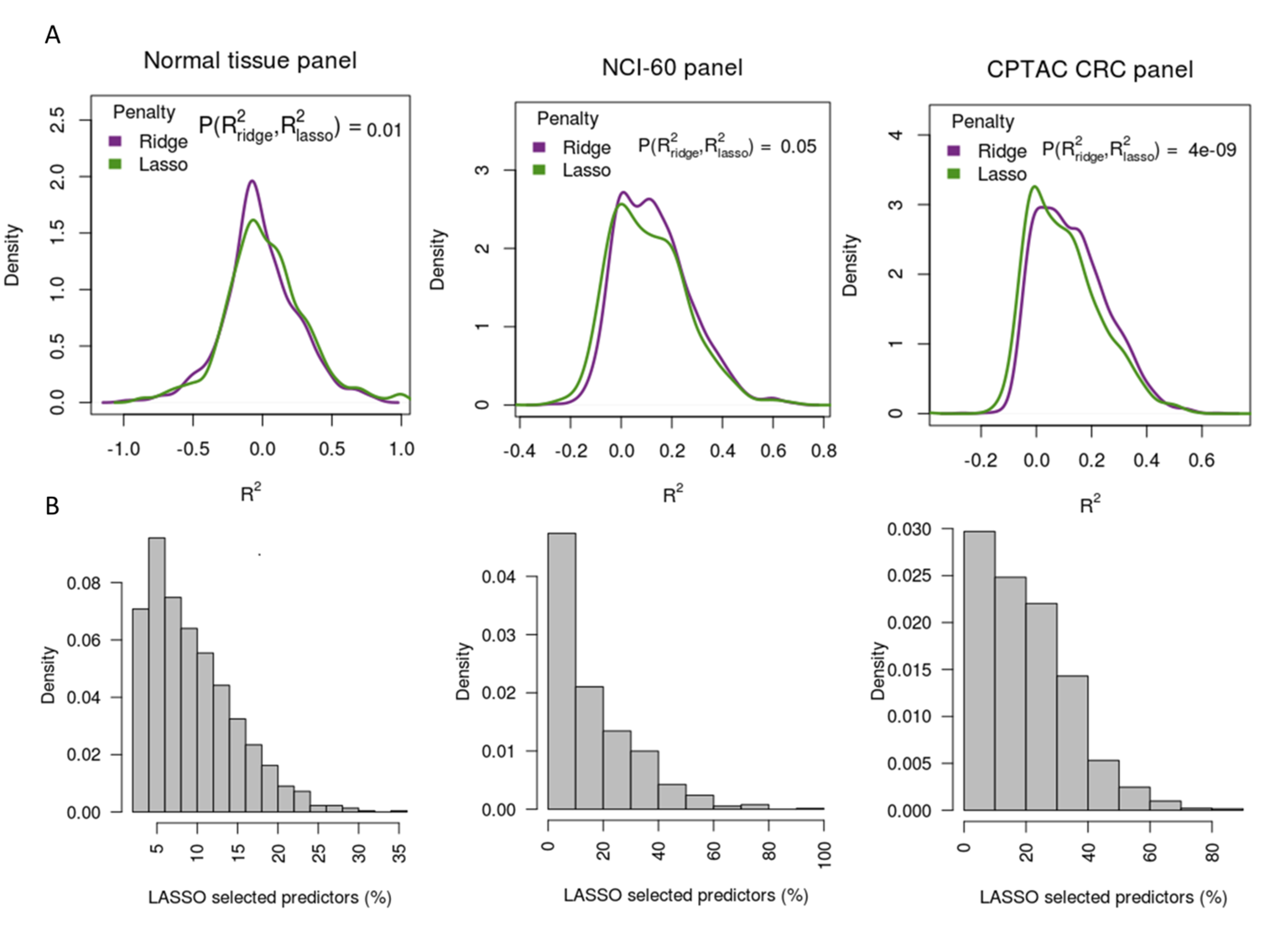

Supplement: S12 Fig — (A) RBPplus models fitted with Ridge or LASSO penalty ensure comparable protein predictive accuracies. Shown are the distributions of R2 obtained by the RBPplus models fitted with Ridge or LASSO penalty. Wilcoxon signed-rank test was used to test differences in rank of the protein predictive accuracy for the RBPplus models fitted by Ridge or LASSO penalty. Test’s P-values are shown. (B) Distribution of the fraction (%) of predictors selected by the RBPplus models fitted with LASSO penalty with respect to the predictors used in the RBPplus models fitted with Ridge penalty. (TIF) [file pcbi.1005198.s012.TIF]

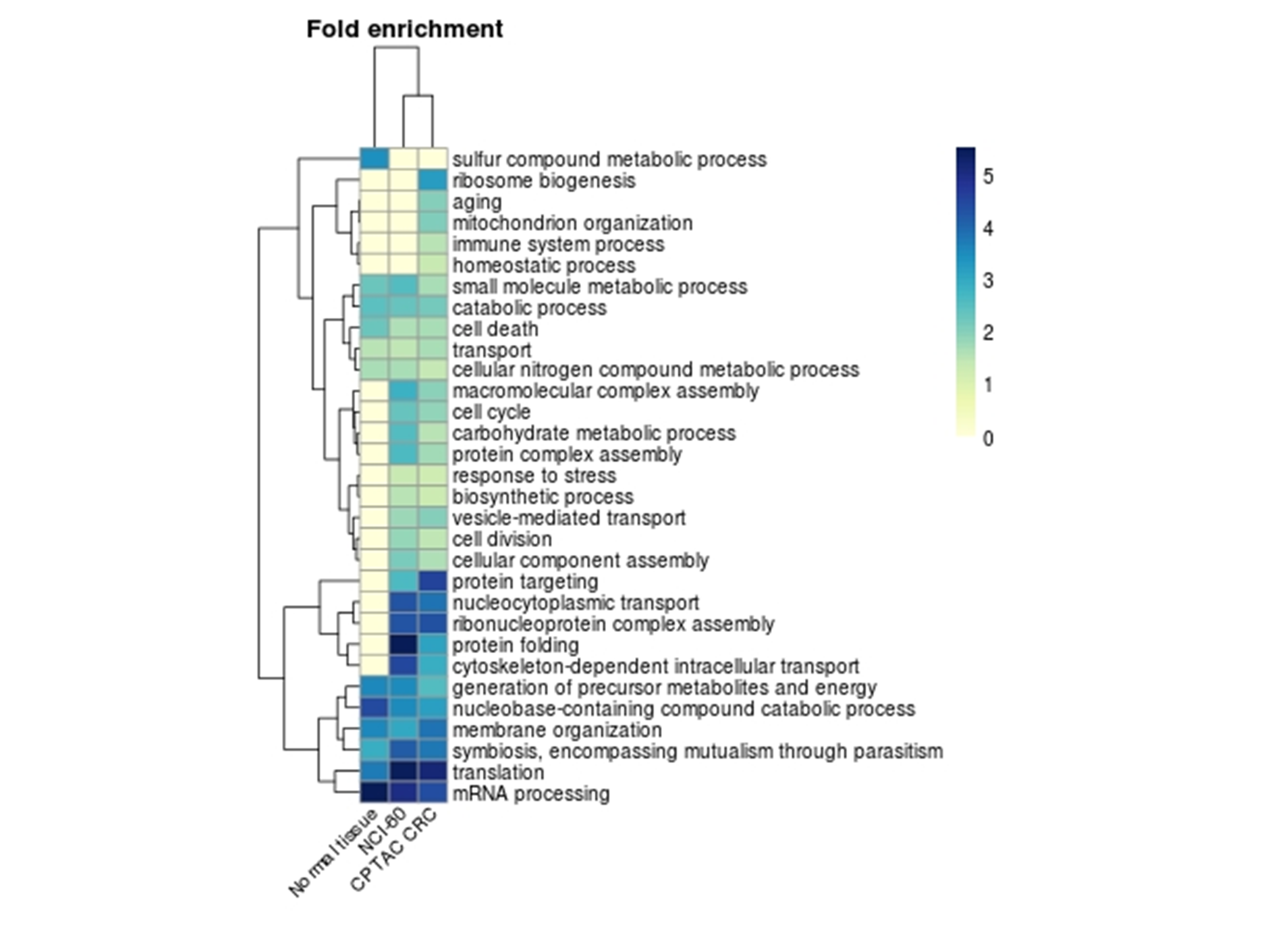

Supplement: S13 Fig — (TIF) [file pcbi.1005198.s013.TIF]

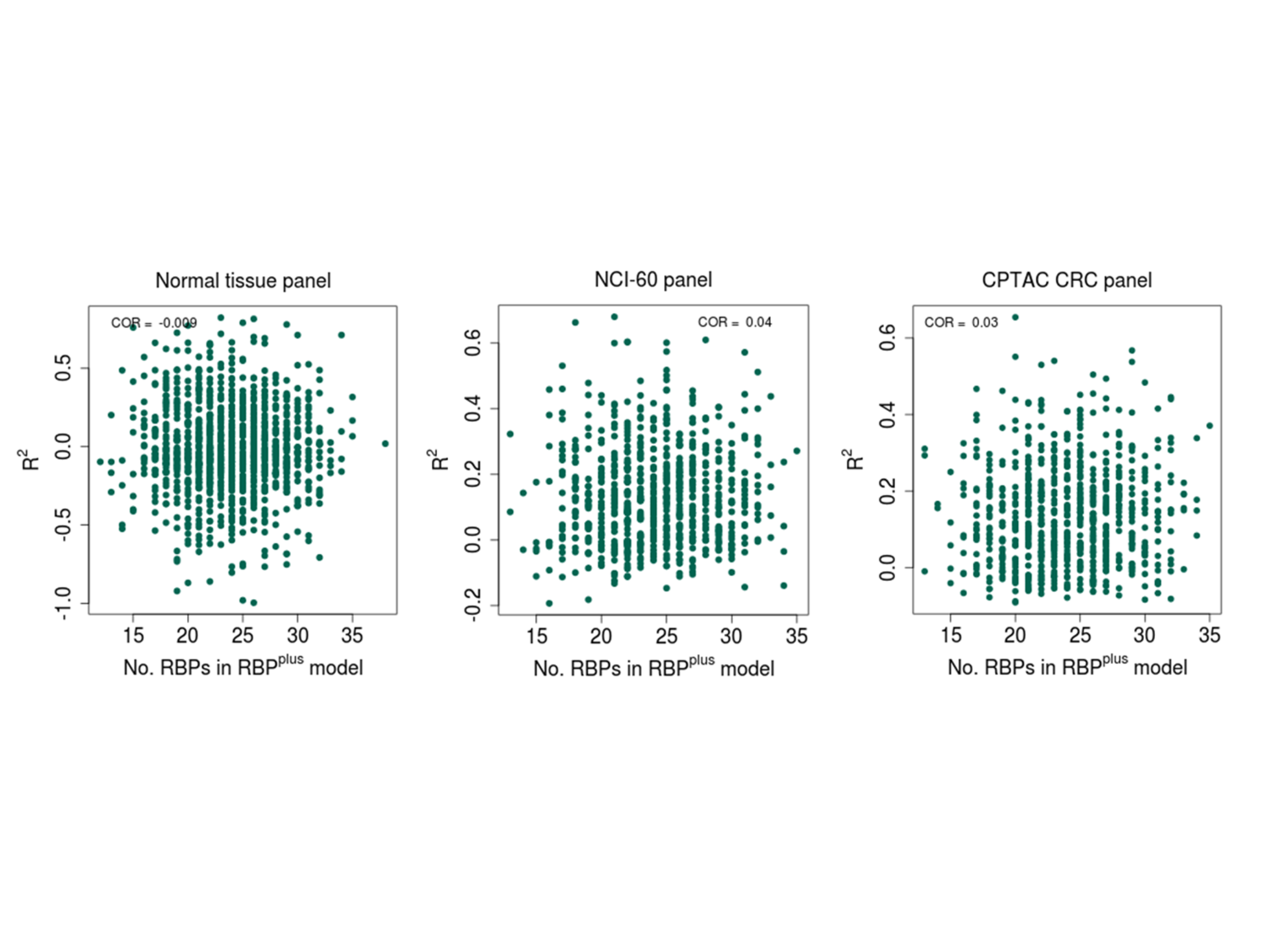

Supplement: S14 Fig — Correlation is estimated by Kendall’s tau coefficient in all three panels. (TIF) [file pcbi.1005198.s014.TIF]

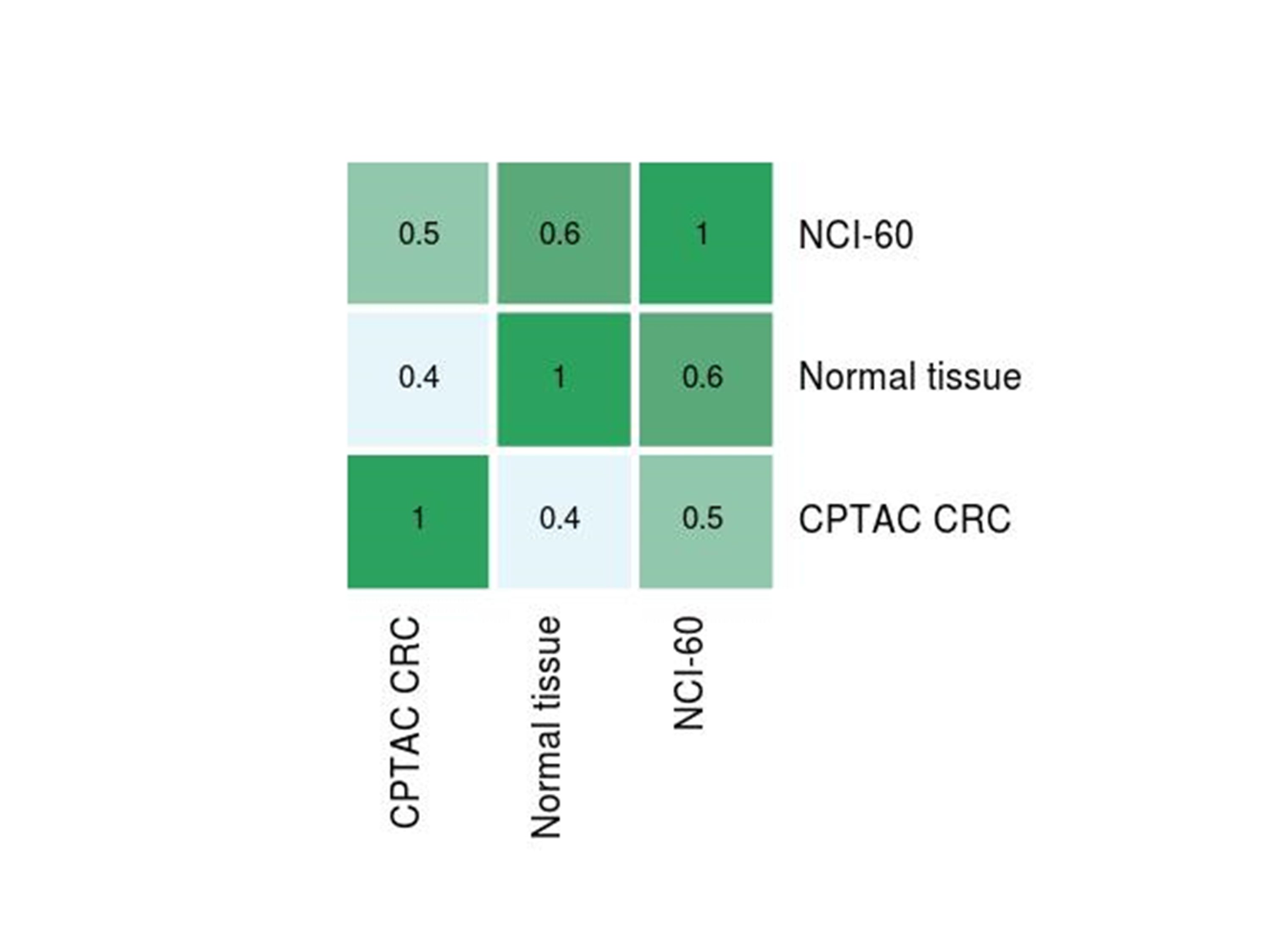

Supplement: S15 Fig — Jaccard index of modelled genes between each pair of panels included in our analysis. (TIF) [file pcbi.1005198.s015.TIF]

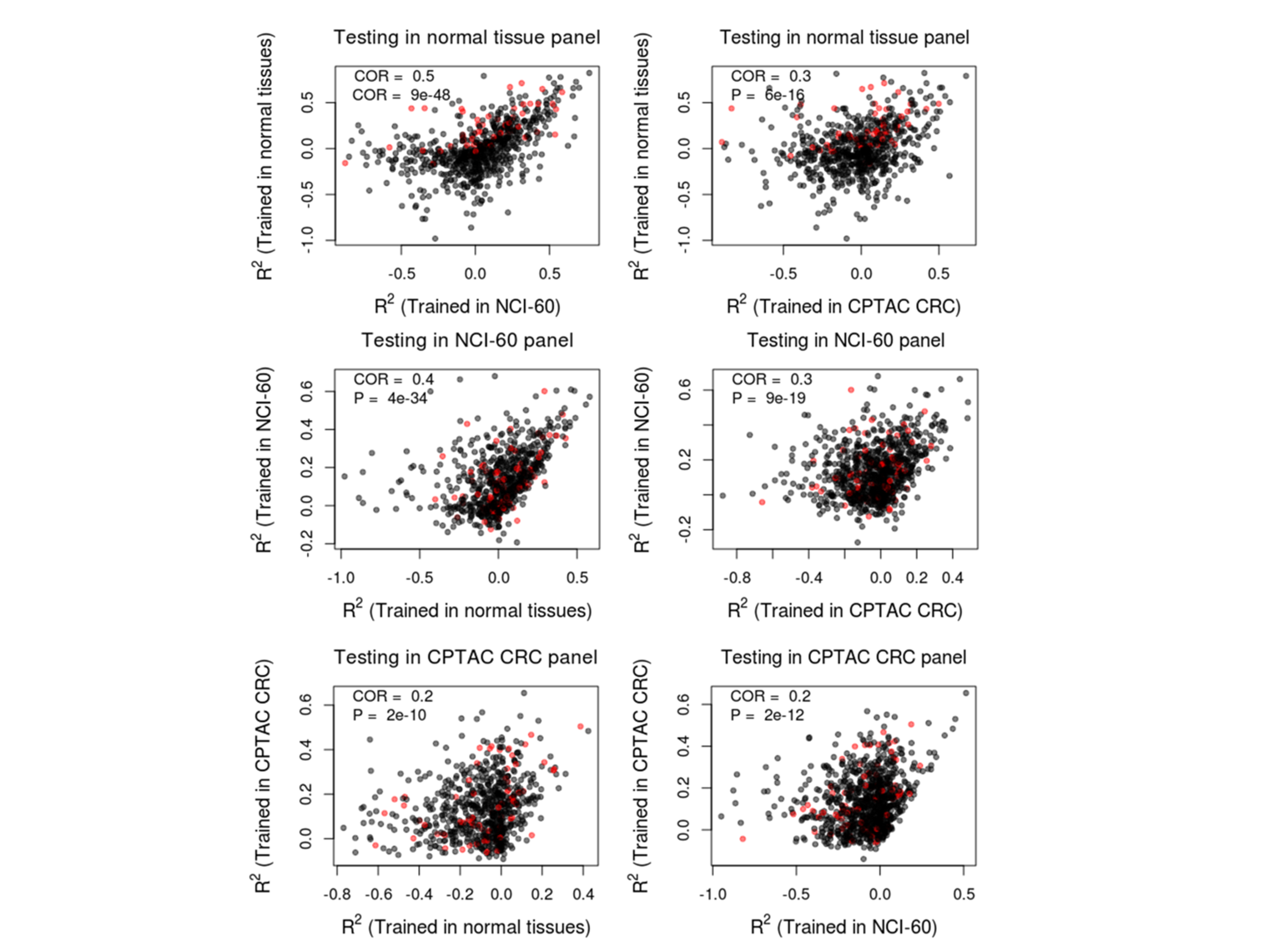

Supplement: S16 Fig — RBPplus models show some transferability across tissue panels. Better transferability is observed from NCI-60 and CPTAC CRC panels to normal tissue panel. All the possible combinations of training and test panels are grouped by test panel. Shown is the Spearman’s correlation coefficient between R2 of RBPplus models trained in the testing panel (shown in vertical axis label) and R2 of RBPplus models trained in the remaining two panels (shown in horizontal axis labels). (TIF) [file pcbi.1005198.s016.TIF]
