# Supplementary material for: Control of Gene Expression by RNA Binding Protein Action on Alternative Translation Initiation Sites
Source: PLoS Comput Biol. 2016 Dec 6;12(12):e1005198. doi: 10.1371/journal.pcbi.1005198 (PMC5140048; doi:10.1371/journal.pcbi.1005198)
Supplement: S1 Text — (DOCX) [file pcbi.1005198.s018.docx]

**Control of gene expression by RNA binding protein action on alternative noncanonical translation initiation sites**

**Angela Re, Levi Waldron, Alessandro Quattrone**

**Data acquisition and processing**

**Gene filtering** Despite recent technological and methodological advancements to achieve high proteome identification and quantification coverage ([(1)](https://paperpile.com/c/zJlF6B/sT27),[(2)](https://paperpile.com/c/zJlF6B/tVNe)), gathering information at the proteome level remains more challenging than at the transcriptome level [(3)](https://paperpile.com/c/zJlF6B/YTM2). Indeed, in each of the matched transcriptome/proteome datasets included in our analysis, the depth of proteomic coverage turned out to lag far behind transcriptome coverage, as shown in **S1 Table**. The relatively low number of genes that was possible for us to model is largely due to this discrepancy between transcriptome/proteome coverages.

A further reduction in the number of genes available for modelling was due to the exclusion of genes below the detection limit in a substantial fraction of samples within each panel. **S1 Fig** displays the fraction of genes by the number of samples where the mRNA and protein levels of the gene were unavailable within each panel.

We avoided genes and proteins that are either not expressed or not reliably measured in the majority of samples. The maximal number of samples without available data at either the transcriptome or proteome level was set to three in the normal tissue panel (which consists of a total of 12 samples) and to five in the NCI-60 and CPTAC CRC panels (which consist of a total of 59 and 87 samples respectively).

Filtering on the basis of availability of proteome data resulted in the selection of more highly expressed proteins with respect to all the proteins detected in at least one of the samples in a panel (**S2 Fig)** because low abundance proteins are in general challenging to detect by quantitative proteomics. This is due both to the low frequency with which low-abundance peptides in complex proteomes are selected for the peptide sequence analysis, which is required for their identification/quantification, and to the fact that signals at the lower bounds of instrument dynamic range barely exceed detection limits.

Finally, we conducted functional enrichment analysis on the genes included in the modelling approach in each panel, using the Gene Ontology GO slim (<http://geneontology.org/page/go-slim-and-subset-guide>/). Hypergeometric test P-values were adjusted for multiple hypotheses testing by the Benjamini-Hochberg procedure. Overall, the genes considered in the modelling analysis turned out to display marginal Gene Ontology depletion, primarily in the normal tissue panel (for developmental maturation, nitrogen cycle metabolic process and cell adhesion), and marginal Gene Ontology enrichment, primarily in the cancer panels for protein folding, ribonucleoprotein complex assembly, and translation (**S3 Fig)**. These are likely to be more highly-expressed proteins, that are thus more easily detected. Fortunately, there are still enough adequately quantitated proteins to provide the diversity in function and genomic features to allow us to investigate whether these features are related to differences in the importance of regulation by RNA-binding proteins.

| **Panel** | **mRNA** | **PROT** |
| --- | --- | --- |
| **Normal tissue** | 20313 | 6104 |
| **NCI-60** | 22825 | 7860 |
| **CPTAC CRC** | 15780 | 7211 |

**Table A.** Transcriptome/proteome coverage in each panel.


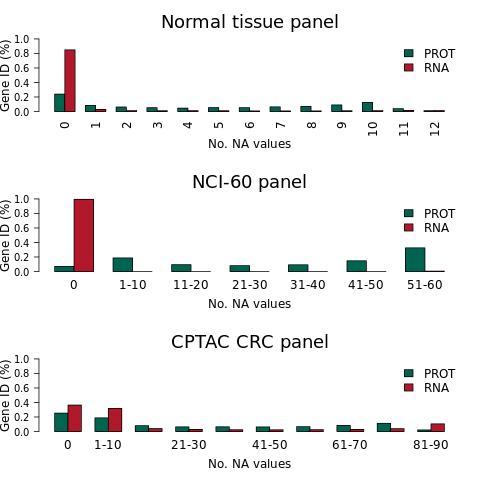


**Supplementary Figure 1.** The fraction of genes is displayed by the number of samples where the mRNA and protein levels of the gene were not detectable.


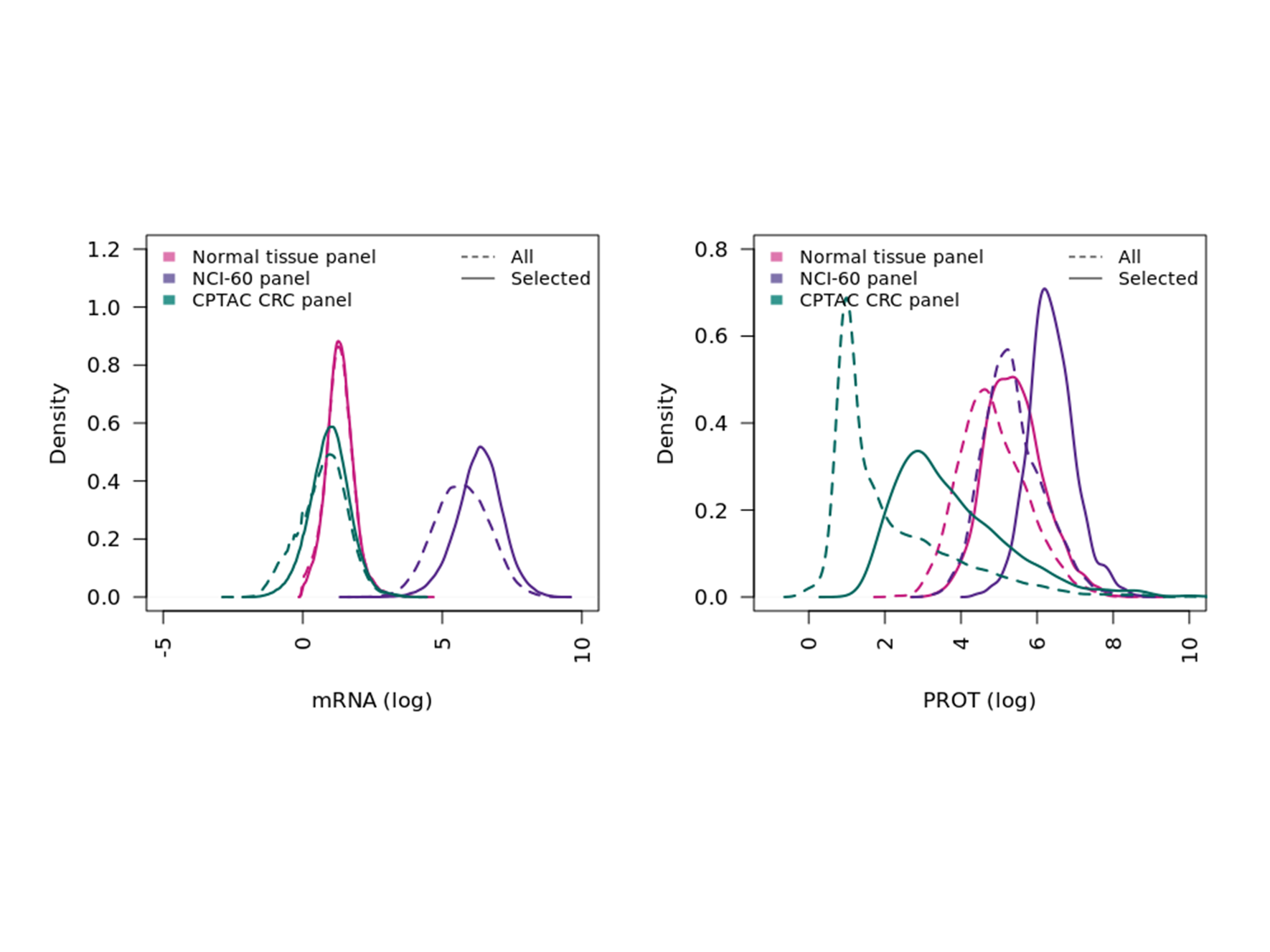


**Supplementary Figure 2.** **Shift in mRNA and protein levels upon gene selection.** (**A**) Distributions of the median logarithmic mRNA abundances of all genes (dashed line) and of the genes selected on the basis of the detection frequency across the samples in each panel (solid line). (**B**) Distributions of the median logarithmic protein abundances of all genes and of the genes selected on the basis of the detection frequency across the samples in each panel.


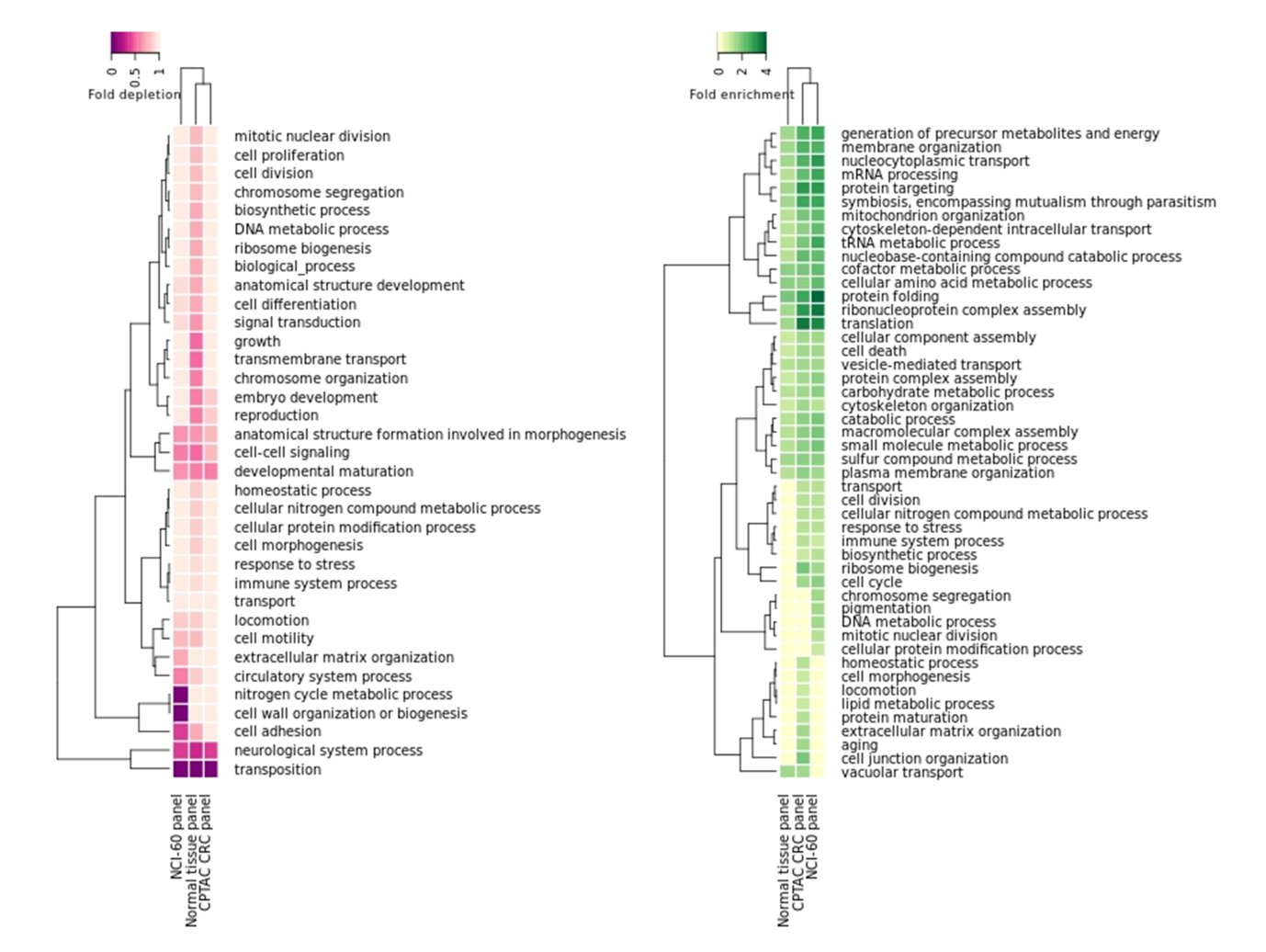


**Supplementary Figure 3. Functional depletion/enrichment in Gene Ontology categories for adequately quantitated genes.** Functional Gene Ontology enrichment analysis of the genes selected for modelling in each panel, showing depleted or enriched GO slim categories (p < 0.05). A Gene Ontology category is shown if false discovery rate meets threshold in at least one panel.

**Inter-sample normalization** Probing complex biological systems, multiple biological and technical factors can interact to produce the variability in average levels which was observed across the samples of each panel.

mRNA expression data turned out not to be originally processed to ensure comparability across samples in any panel (**S4 Fig**).


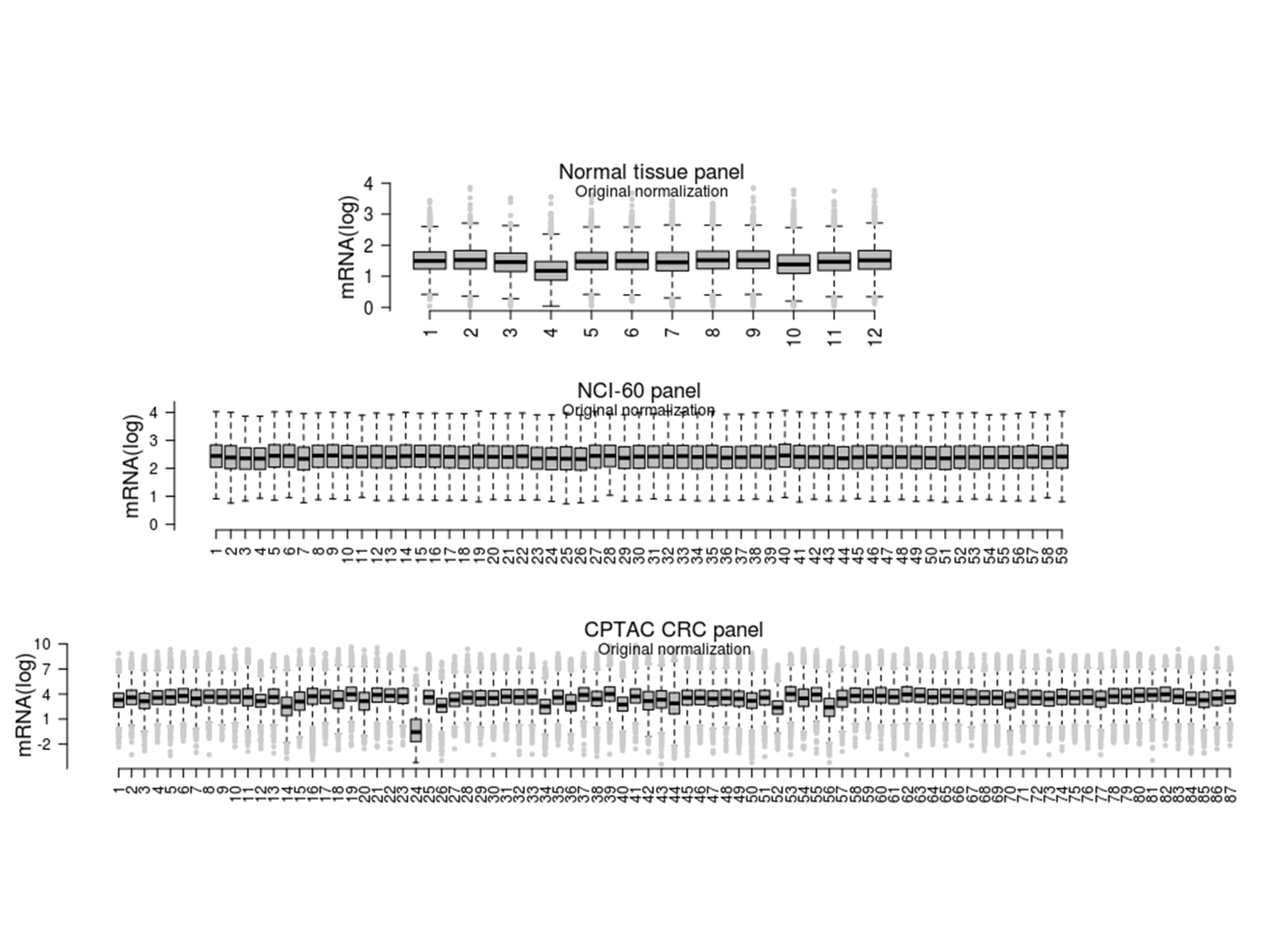


**Supplementary Figure 4 mRNA abundance quantification in each panel.** mRNA expression data are unmodified with respect to the original publication. **(A)** Distribution of Fragments Per Kilobase per Million (FPKM) from RNA-seq experiments of all 12 normal tissue samples. **(B)** Distribution of mRNA intensity from microarray profiling experiments of all 59 NCI-60 cell lines. **(C)** Distribution of Fragments Per Kilobase per Million (FPKM) from RNA-seq experiments of all 87 CPTAC CRC samples.

Protein levels in the normal tissue and NCI-60 panels turned out to be more affected by inter-sample variability than the CPTAC CRC panel. This was due to a proper inter-sample normalization scheme (quantile normalization) in the protein expression data analysis in the CPTAC CRC panel, which lacked in normal tissue and NCI-60 panels. Note that the distributions of CPTAC CRC protein levels depicted in **S5 Fig** are not identical across samples since we plotted the expression data corresponding just to the genes selected for our modelling approaches.

**
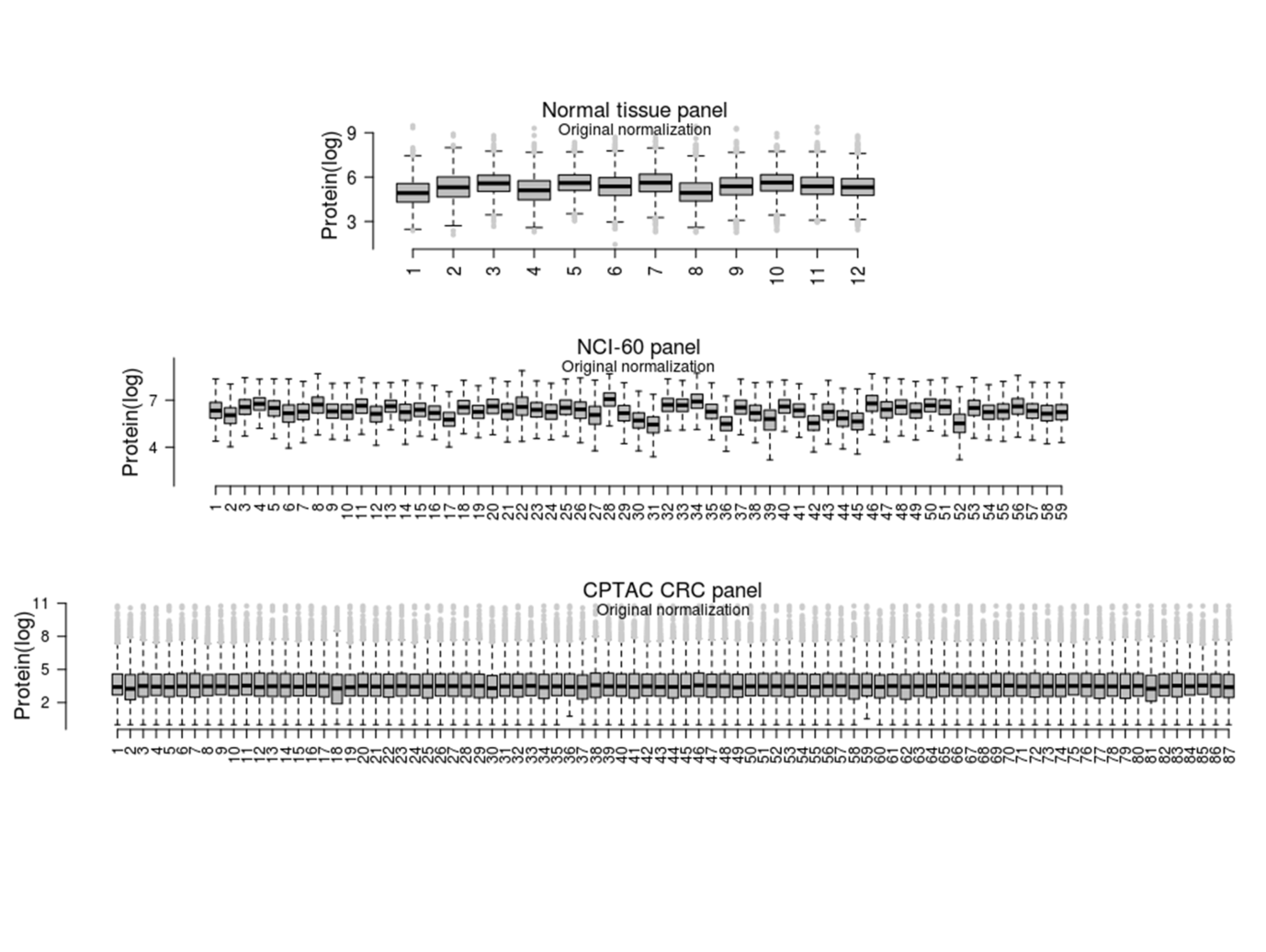
**

**Supplementary Figure 5** **Protein abundance quantification in each panel.** Protein expression data are unmodified with respect to the original publication. **(A)** Distribution of protein intensity from proteome profiling experiments of all 12 normal tissue samples. **(B)** Distribution of protein intensity from proteome profiling experiments of all 59 NCI-60 cell lines. **(C)** Distribution of spectral counts from proteome profiling experiments of all 87 CPTAC CRC samples.

As a result of variability in average levels, the expression of random proteins could help in predicting protein levels. To eliminate this possibility, we investigated two approaches to normalize the mRNA and protein expression data across the samples of each panel: (1) subtraction of average mRNA and protein expression in each sample, and (2) quantile normalization. The former approach eliminates variability in protein expression distributions across samples which can be due to different sample averages, while the latter approach makes the distributions of expression levels identical across samples. Note that we applied normalization by subtraction of average mRNA and protein levels per sample but we did not apply quantile normalization to protein levels which were already normalized.

Data normalization across samples generally caused an improvement in protein predictive accuracy for the RNA^only^ models and a reduction for the RBP^plus^ models (**S6 Fig**). As expected, the effect was barely noticeable in the CPTAC CRC panel which was already quantile normalized, whereas it was substantial in the NCI-60 panel. Nevertheless, the predictive accuracy of RBP^plus^ models was confirmed to be statistically significantly higher than the accuracy of RNA^only^ models upon either quantile normalization or normalization by mean-centring per sample (Wilcoxon signed-rank test, p < 0.05).

This analysis showed that inter-sample normalization was necessary to draw reliable conclusions from the regression approaches. Unless stated otherwise, the expression data used to develop our regression approaches are meant to be normalized by subtraction of average protein level per sample.

**
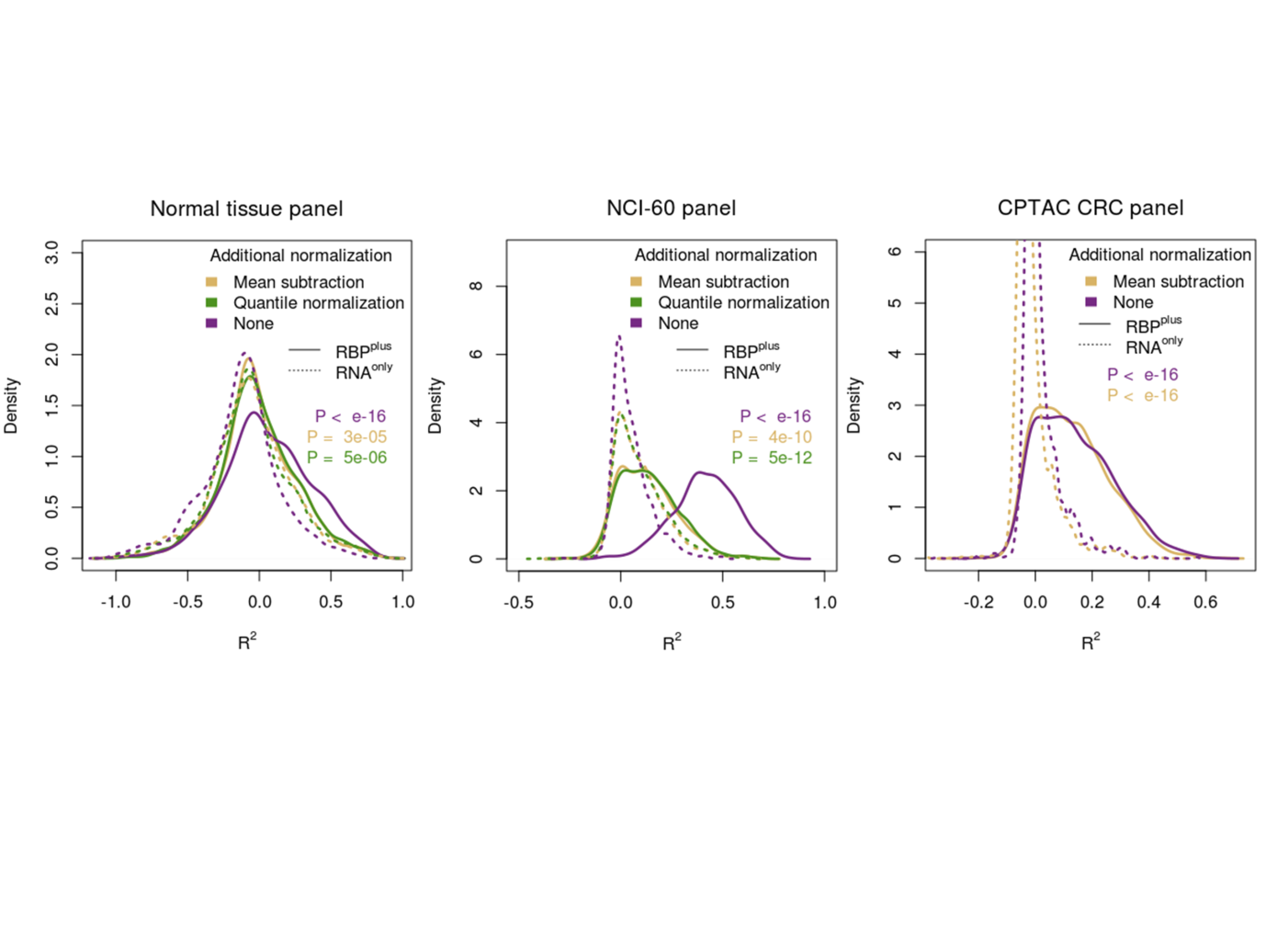
**

**Supplementary Figure 6 Inter-sample normalization effects on model performances.** Distribution of R^2^ achieved by the RNA^only^ (dashed line) and RBP^plus^ (solid line) models according to different types of inter-sample normalization. Shown are p-values of Wilcoxon signed-rank tests to assess differences in the ranks of predictive accuracies between the RNA^only^ and the RBP^plus^ models based on each type of inter-sample normalization.

**Analysis of influential observations.** A sample is influential on a regression model if it has an unusual value of the predictor variable (leverage) and an unusual value of the dependent variable conditional on the value of the predictor variable (discrepancy). Cook’s distance measures both leverage and discrepancy. Consequently, we used the Cook’s Distance to measure the influence of every sample in a simple linear regression model of each gene considered in each panel. **S7** **Fig** shows that influential observations were very sparse and no sample turned out to be systematically associated with influential observations.


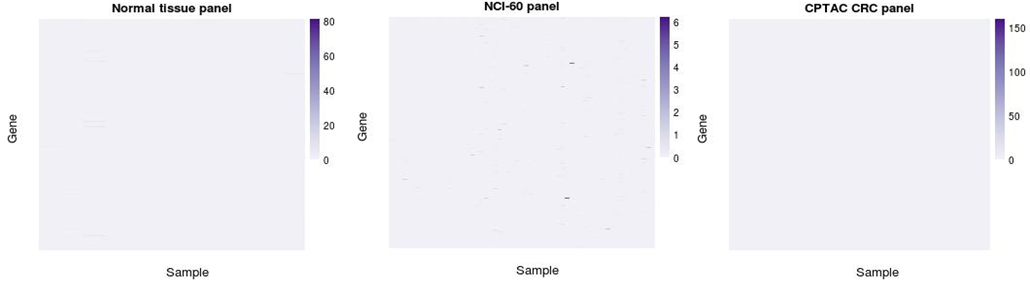


**Supplementary Figure 7 Influential observations are sparse in all the three panels.** Heat maps display Cook’s distance values for each gene and sample.

**Regulation of mRNAs by RNA binding proteins is combinatorial**

We summarized the picture emerging from the RBP binding site inferential process by displaying the distribution of the number of mRNAs where the UTRs were inferred to be bound by an RBP and the distribution of RBPs which were inferred to bind the UTRs of an mRNA. Even if the number of RBPs inferred to bind an mRNA usually decreased when lowering the threshold to the FDR on the inferred RBP binding sites from 20% to 5%, we generally found that multiple mRNAs were inferred to be bound by multiple RBPs (**S8 Fig**).

**
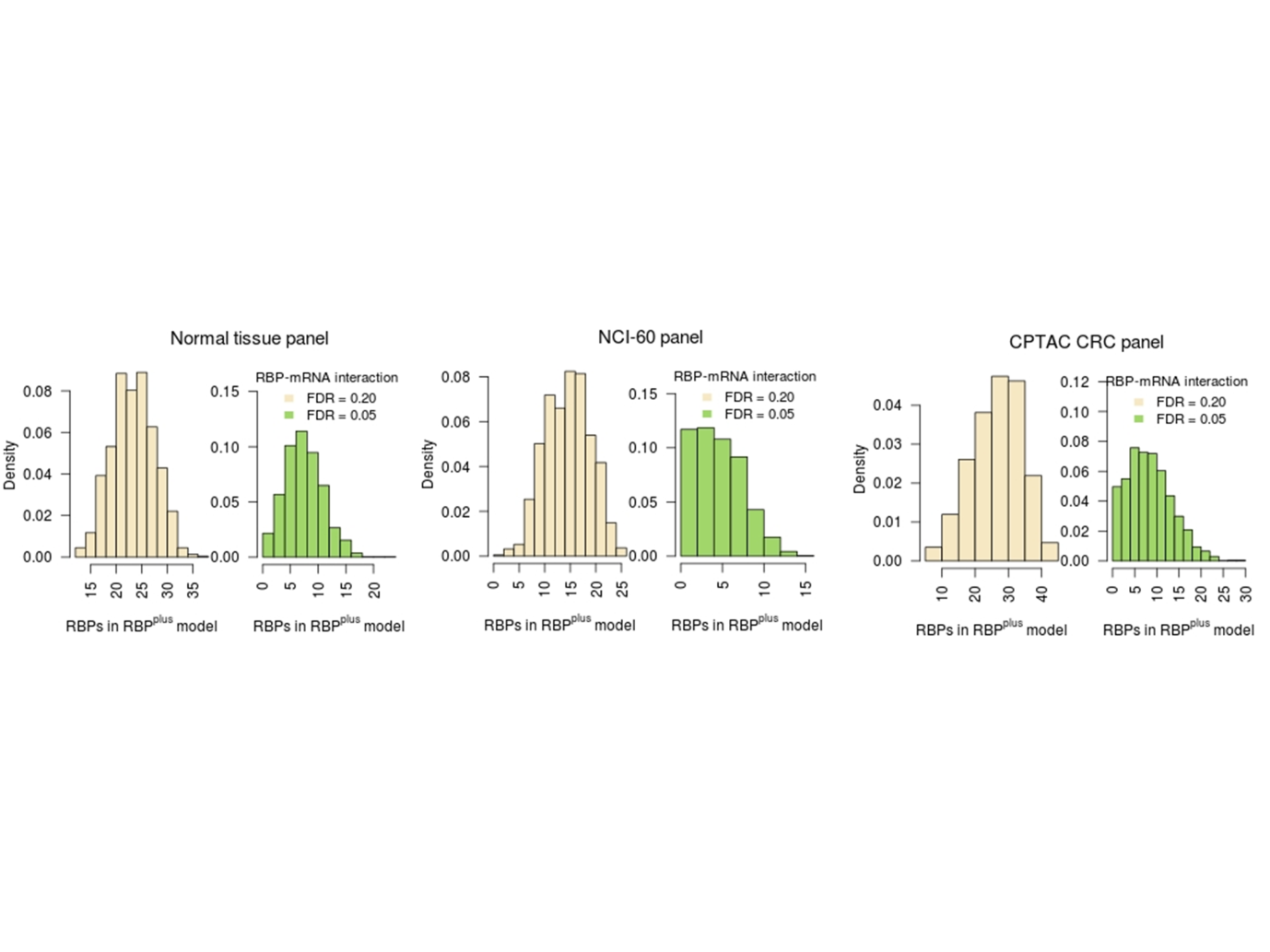
**

**Supplementary Figure 8 Predicted RBP-mRNA interactions are combinatorial.** Distribution of number of RBPs inferred per mRNA using the thresholds of 5% or 20% to the false discovery rate on RBP binding sites.

**Clustering analysis of the network of inferred interactions between RNA binding proteins and mRNAs**

Network analysis was aimed at identifying the genes where the RBP^plus^ models afforded large improvement in predictive accuracy as well as the RBPs which showed relatively great regression coefficients in the RBP^plus^ models of those genes.

First, we framed the collection of inferred RBP-mRNA interaction into a directed and weighted network where source nodes were the RBPs, target nodes the mRNAs, and where edges were drawn if the RBPs were inferred to bind the mRNAs.

Second, we applied the following network weighting scheme. The weight of a target (mRNA) node was defined by the RMSE ratio between the RNA^only^ and RBP^plus^ models of the mRNA. Source nodes received weights equal to 1. The weight of an RBP-mRNA edge was defined by the absolute value of the regression coefficient that the RBP showed in the RBP^plus^ model of the mRNA.

Third, we applied a clustering algorithm to the aforementioned weighted network in order to identify clusters of highly predictive RBP-mRNA interactions. A highly predictive RBP-mRNA interaction is an interaction where the RBP obtains a large regression coefficient in the RBP^plus^ model of the mRNA and the RBP^plus^ model affords a large improvement in predictive accuracy relative to the RNA^only^ model. Since the information related to the RBP^plus^ models (improvement in predictive accuracy, RBP regression coefficient) is encoded in the network weights, we adopted the Weighted Graph Clustering approach (WG-Cluster) algorithm, which uses both network node and edge weights for cluster identification [(4)](https://paperpile.com/c/zJlF6B/yflc). WG-Cluster utilizes three major steps: (i) an unsupervised version of k-means edge-based algorithm detects sub-graphs with similar edge weights, (ii) a fast-greedy algorithm detects connected components which are then given an entropy score reflecting the weights and the weighted degrees of the constituent nodes, and (iii) a randomization-based procedure computes the statistical significance of their scores.

Six connected components were selected by requiring that they totalized an entropy score and a mean edge weight of the sub-graph higher than the median values overall detected connected components (**S9A Fig**). As expected, the protein levels predicted by the RBP^plus^ model on the clustered mRNAs were found to be generally closer to the experimentally determined protein levels, when compared to the protein levels predicted by the RNA^only^ model (Wilcoxon-Mann-Whitney test, P-value < 0.05, **S9B Fig**). Network clustering analysis was complemented with Gene Ontology based functional enrichment analysis to evaluate the collective extent of shared functionalities within and across modules. A large number of genes were found to localize in the mitochondrion and to encode protein implicated in energy derivation metabolism. Another interesting group of genes observed in multiple modules were protein chaperones subunits of the TCP1 ring complex (TRiC), which folds essential and topologically complex proteins including cell-cycle regulators, signalling proteins and cytoskeletal components.

**
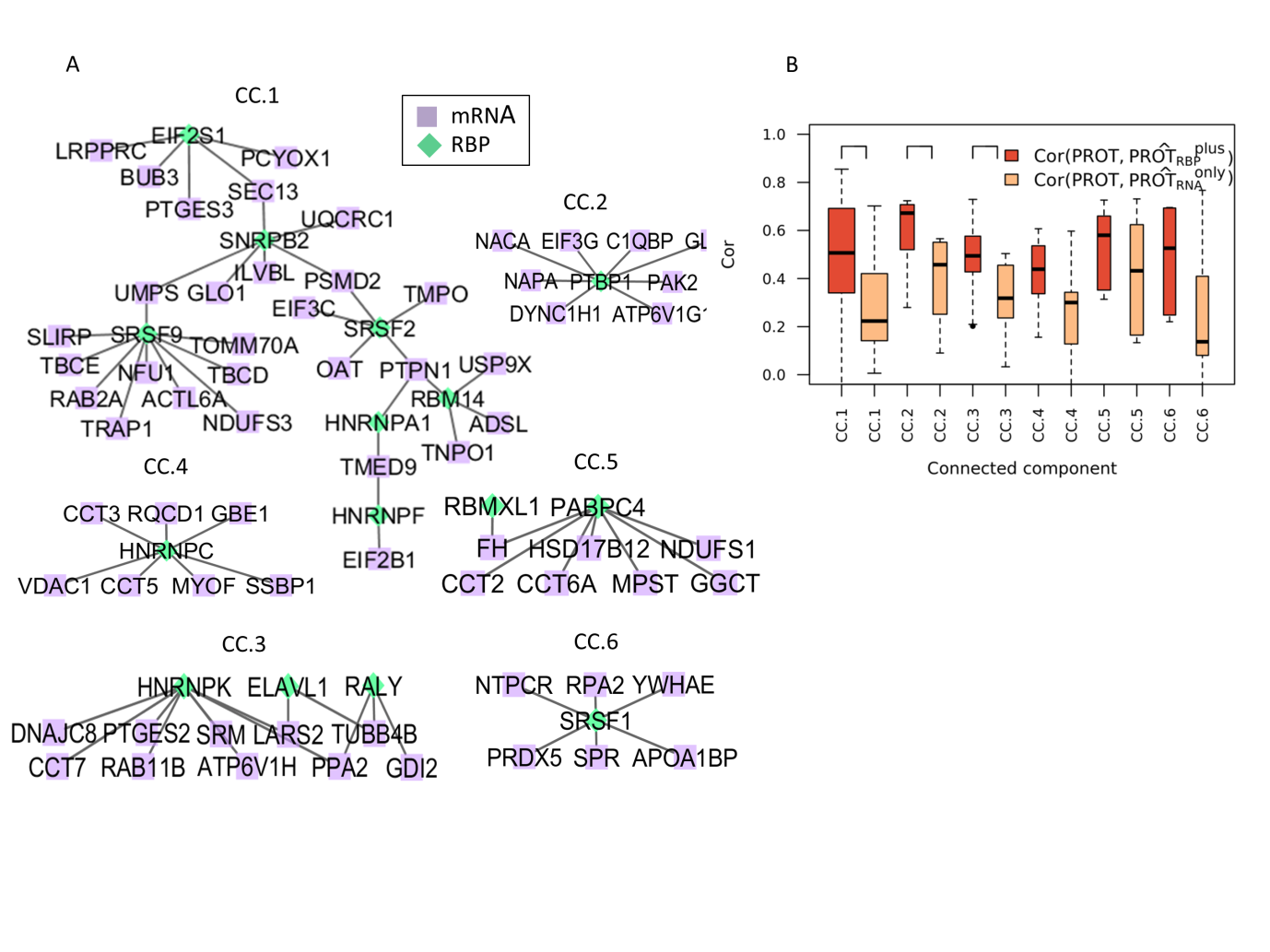
**

**Supplementary Figure 9 Network clustering analysis delivers modules of RBP-RNA interactions yielding improvement in protein prediction accuracy.** (**A**) Node colour distinguishes source (RBP predictor) and target (modelled gene) nodes. An edge indicates that the RBP is predicted to bind the mRNA. A target node weight is introduced to represent the improved accuracy in the protein abundance prediction of the RBP^plus^ model in comparison to the RNA^only^ one, whereas an edge weight represents the regression coefficient of the RBP in the RBP^plus^ model of the target mRNA. Only statistically significant modules totalizing mean edge weight and entropy values above median values are displayed. (**B**) Gene-wise correlations between experimental protein levels and protein levels predicted, respectively, by the RBP^plus^ and the RNA^only^ models are shown for each module. The RBP^plus^ model improves the correlation between inferred and observed protein levels in all modules. The modules where the improvement is statistically significant display pincers on the top of the corresponding pairs of boxplots.

**Sensitivity analysis**

Using the more stringent threshold of 5% to the FDR on RBP binding site predictions, RBP^plus^ models were confirmed to improve predictive accuracy over RNA^only^ models in the normal tissues and CPTAC CRC panels but not in the NCI-60 panel (**S10 Fig**).

**
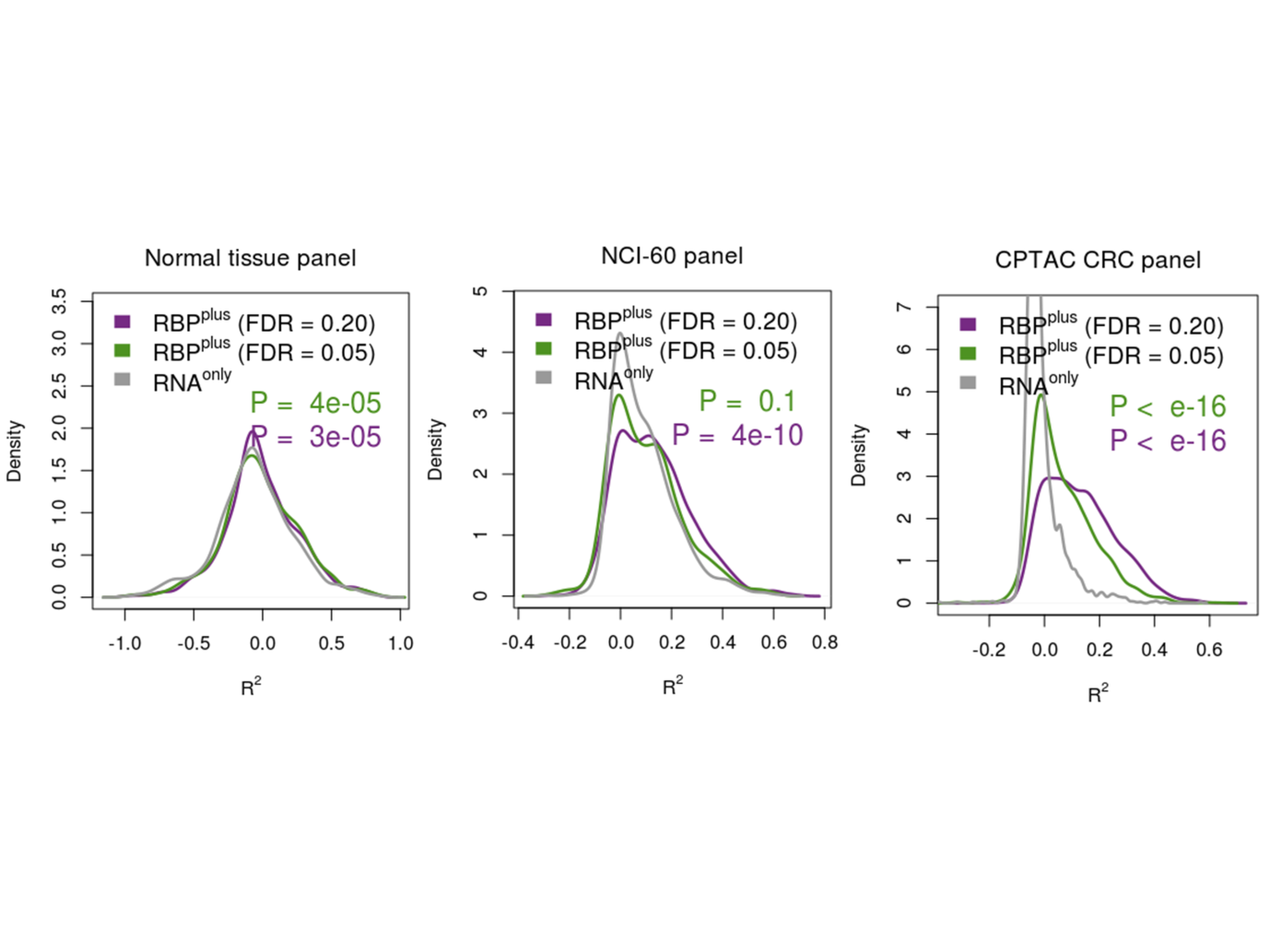
**

**Supplementary Figure 10 Improvement of RBP^plus^ model relative to RNA^only^ model is independent of stringency to infer RBP-mRNA interactions.** Shown are the distributions of protein predictive accuracy (R^2^ ) obtained by the RNA^only^ models as well as by the RBP^plus^ models using RBP-mRNA interactions inferred at different false discovery rates (FDRs). We tested differences in rank of protein predictive accuracies between RNA^only^ models and RBP^plus^ models at different FDR values by the Wilcoxon signed-rank test. P-values are shown and colour-coded in figure.

**Comparison of penalized regression by Ridge and LASSO penalty**

LASSO penalized RBP^plus^ models were fitted with the same procedure penalized regression with Ridge penalty. Penalized RBP^plus^ models with LASSO and penalty for each mRNA/protein pair were fit using RNA level as an unpenalized covariate and protein levels as penalized covariates to help control overfitting. We used the implementation of LASSO penalized linear regression by nested cross-validation which is available in the function *opt.nested.crossval* of the R package *pensim*. In the outer level of cross-validation, samples were split into training and test samples. Cross-validation within training of samples only was used to tune the LASSO and Ridge penalty parameters.

We tested the differences in rank of protein predictive accuracies for the LASSO penalized RBP^plus^ and the RNA^only^ models (Wilcoxon signed-rank test). We found that RBP^plus^ models fitted by LASSO ensured statistically significantly higher predictive accuracy relative to the RNA^only^ models (p < 0.05, **S11 Fig**).


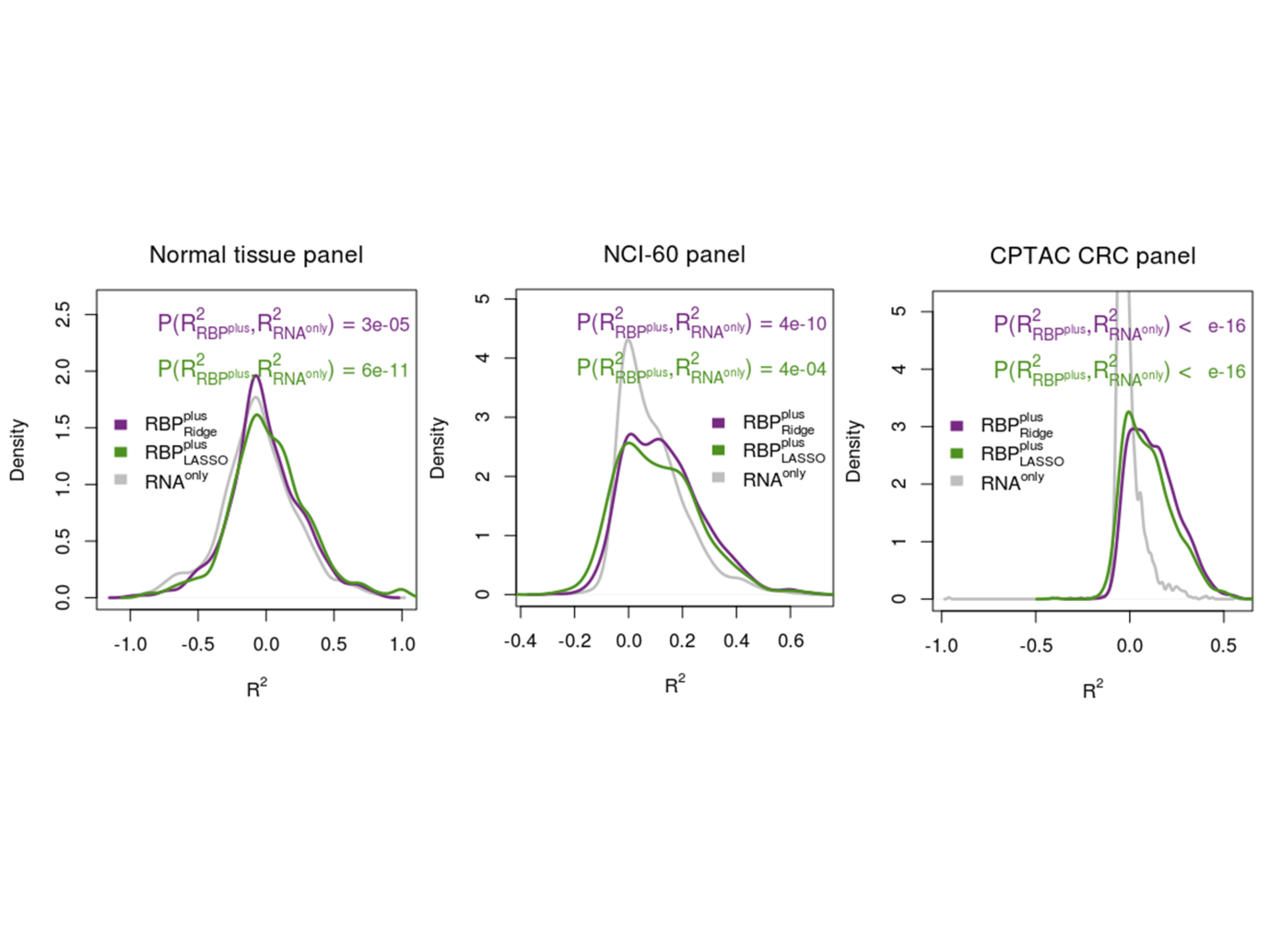


**Supplementary Figure 11 RBP^plus^ models fitted by LASSO ensure better protein predictive accuracy relative to the RNA^only^ models.** The distributions of protein predictive accuracy (R^2^) for the RBP^plus^ models fitted with Ridge and LASSO penalty are shown with the R^2^ distribution for the RNA^only^ models. Wilcoxon signed-rank test was used to test differences in rank of the protein predictive accuracy for the RNA^only^ models and the RBP^plus^ models, which were fitted by either penalty. Test’s P-values are colour-coded according to the penalty used to fit RBP^plus^ models.

Furthermore, LASSO penalized RBP^plus^ models were found to be equivalent to Ridge penalized RBP^plus^ models in protein predictive accuracy in two out of three panels (Wilcoxon signed-rank test, p < 0.05, **S12 Fig**).

**
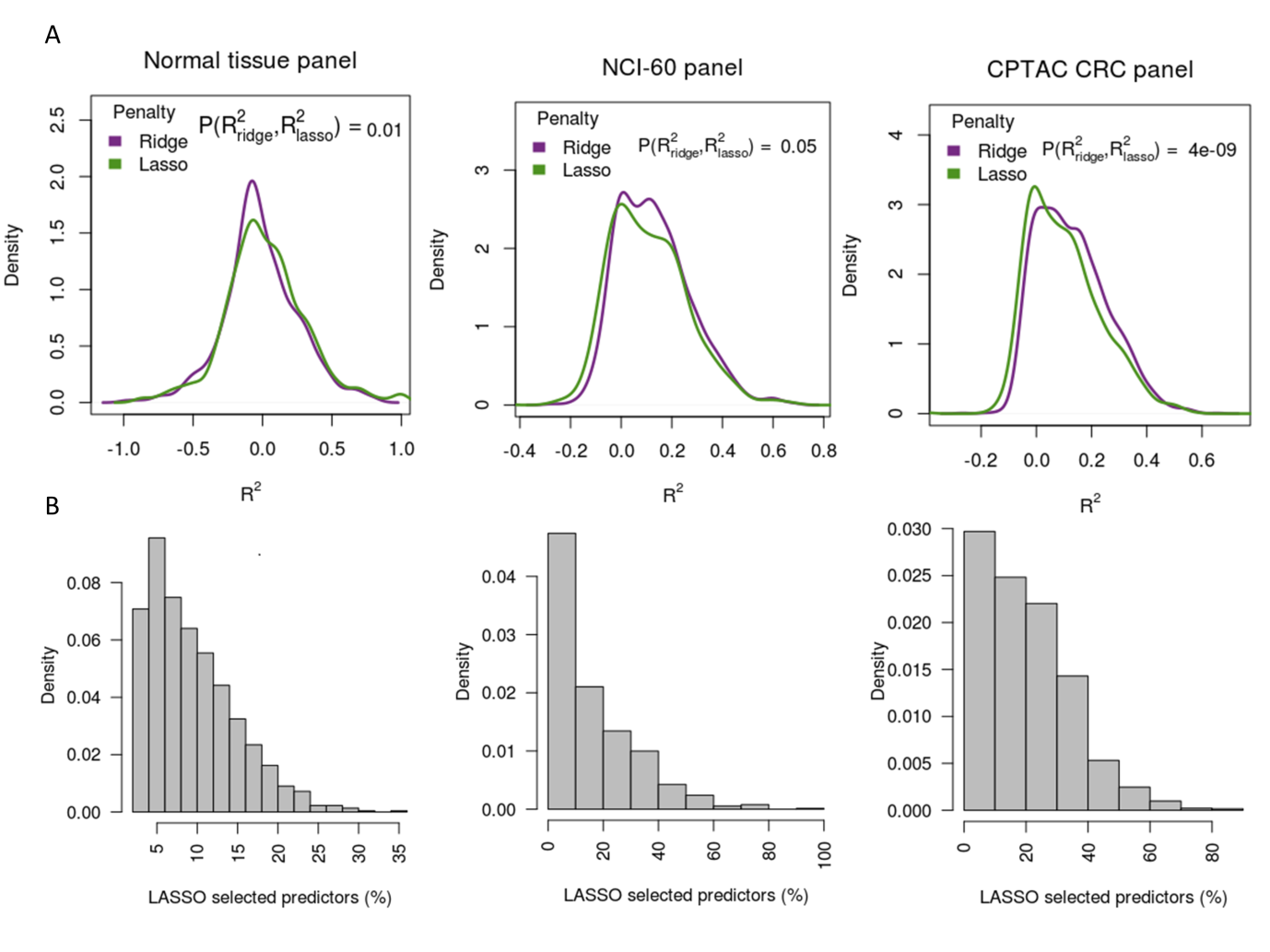
**

**Supplementary Figure 12** **(A)** RBP^plus^ models fitted with Ridge or LASSO penalty ensure comparable protein predictive accuracies. Shown are the distributions of R^2^ obtained by the RBP^plus^ models fitted with Ridge or LASSO penalty. Wilcoxon signed-rank test was used to test differences in rank of the protein predictive accuracy for the RBP^plus^ models fitted by Ridge or LASSO penalty. Test’s P-values are shown. **(B)** Distribution of the fraction (%) of predictors selected by the RBP^plus^ models fitted with LASSO penalty with respect to the predictors used in the RBP^plus^ models fitted with Ridge penalty.

**Functional enrichment analysis**

We performed functional Gene Ontology enrichment / depletion analysis of genes where the RBPplus model achieved statistically significantly better protein predictive accuracy than expected for RBPplus models randomized by randomly sampling protein predictors (FDR < 0.05). We identified enrichment of these genes in several categories but no depletion (**S13 Fig**). We noticed that the most highly overrepresented categories pointed at the involvement of these genes in mRNA processing and translation across all the panels.


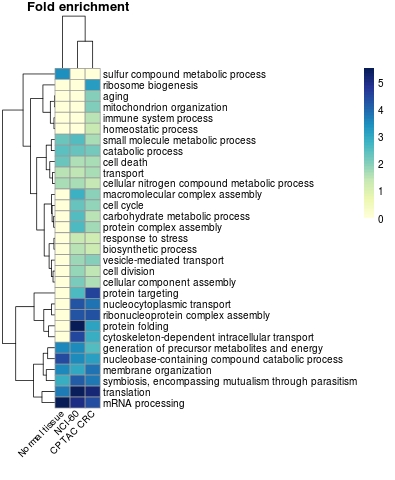


**Supplementary Figure 13** Gene Ontology categories in the Biological Process domain overrepresented (p < 0.05) in genes where the RBP^plus^ model achieved statistically significantly better protein predictive accuracy than expected for RBP^plus^ models randomized by randomly sampling protein predictors.

**Robustness analysis**


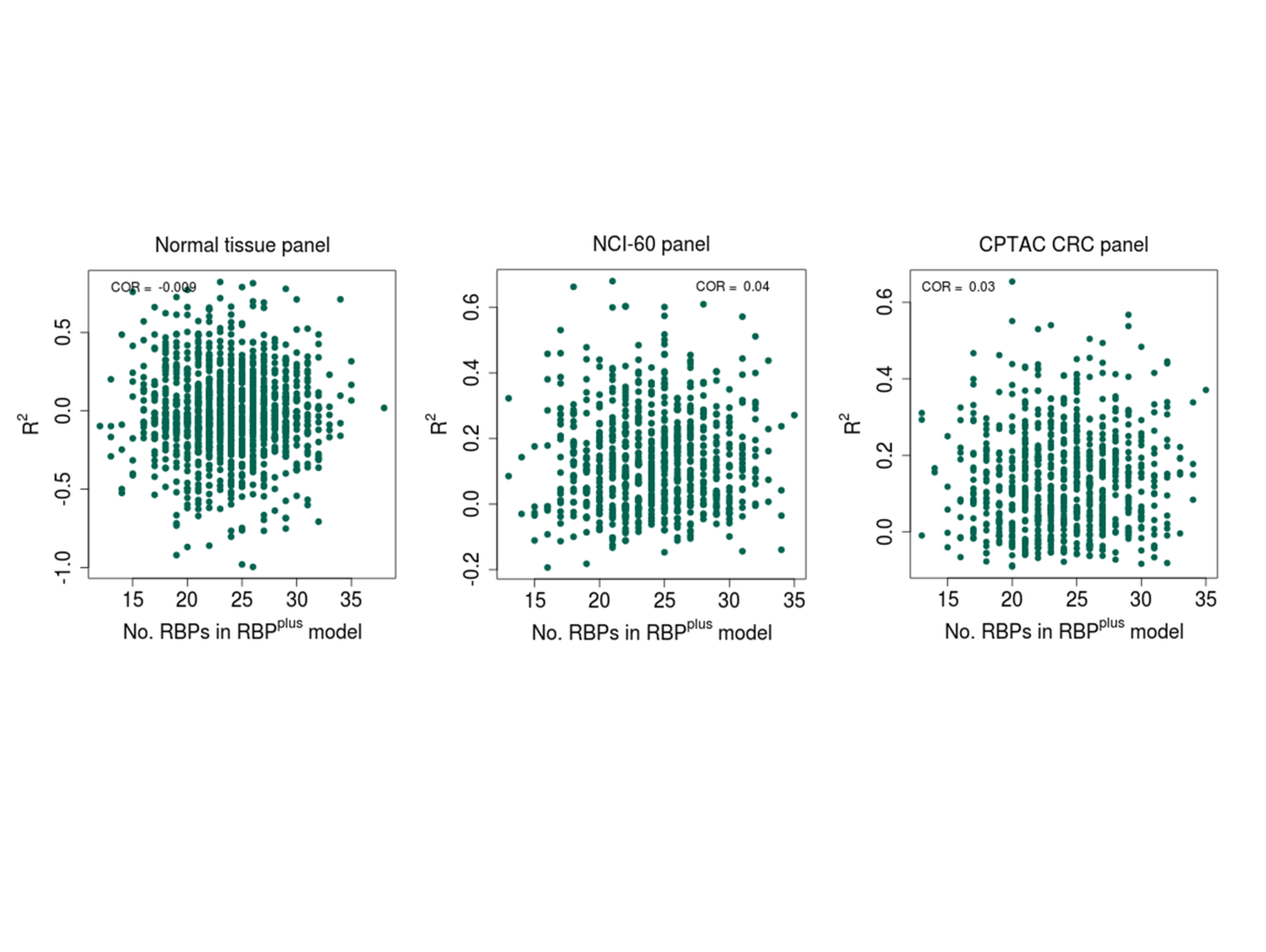


**Supplementary Figure 14 Protein predictive accuracy (R^2^) and number of RBPs in the RBP^plus^ models do not correlate.** Correlation is estimated by Kendall’s tau coefficient in all three panels.

**Cross-panel model transferability**

Protein coverage was found to be different across the three panels (**S13 Fig**), substantially limiting the number of proteins available for cross-panel transfer of models.


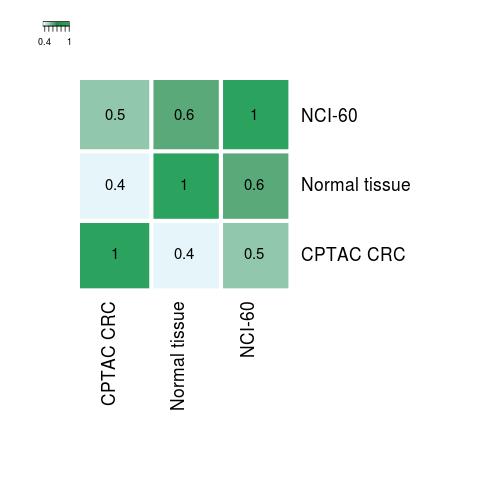


**Supplementary Figure 15 Overlap of modelled genes across panels.** Jaccard index of modelled genes between each pair of panels included in our analysis.

We studied cross-panel model transferability of models trained using only the RBPs profiled in both of each pair of panels. For each considered mRNA, we developed the RNA^only^ and RBP^plus^ models using all samples in a training panel, and tested them using all samples in the testing panel. The procedure was repeated for all possible combinations of training and test panel. We estimated model transferability computing Spearman’s correlation coefficient of protein predictive accuracies between the RBP^plus^ models trained in a chosen panel and the RBP^plus^ models trained in each of the other two panels. Despite the uneven protein coverage and the heterogeneous nature of the training/test panels, correlation reached statistical significance, ranging from 0.2 to 0.5 depending of the combination of train/test panels (**S16 Fig**). We noticed that better correlation were observed when RBP^plus^ models were trained in the NCI-60 and CPTAC CRC panels and transferred to the normal tissue panel, possibly due to the limitations of training accurate models in only 12 samples of the normal tissue panel.


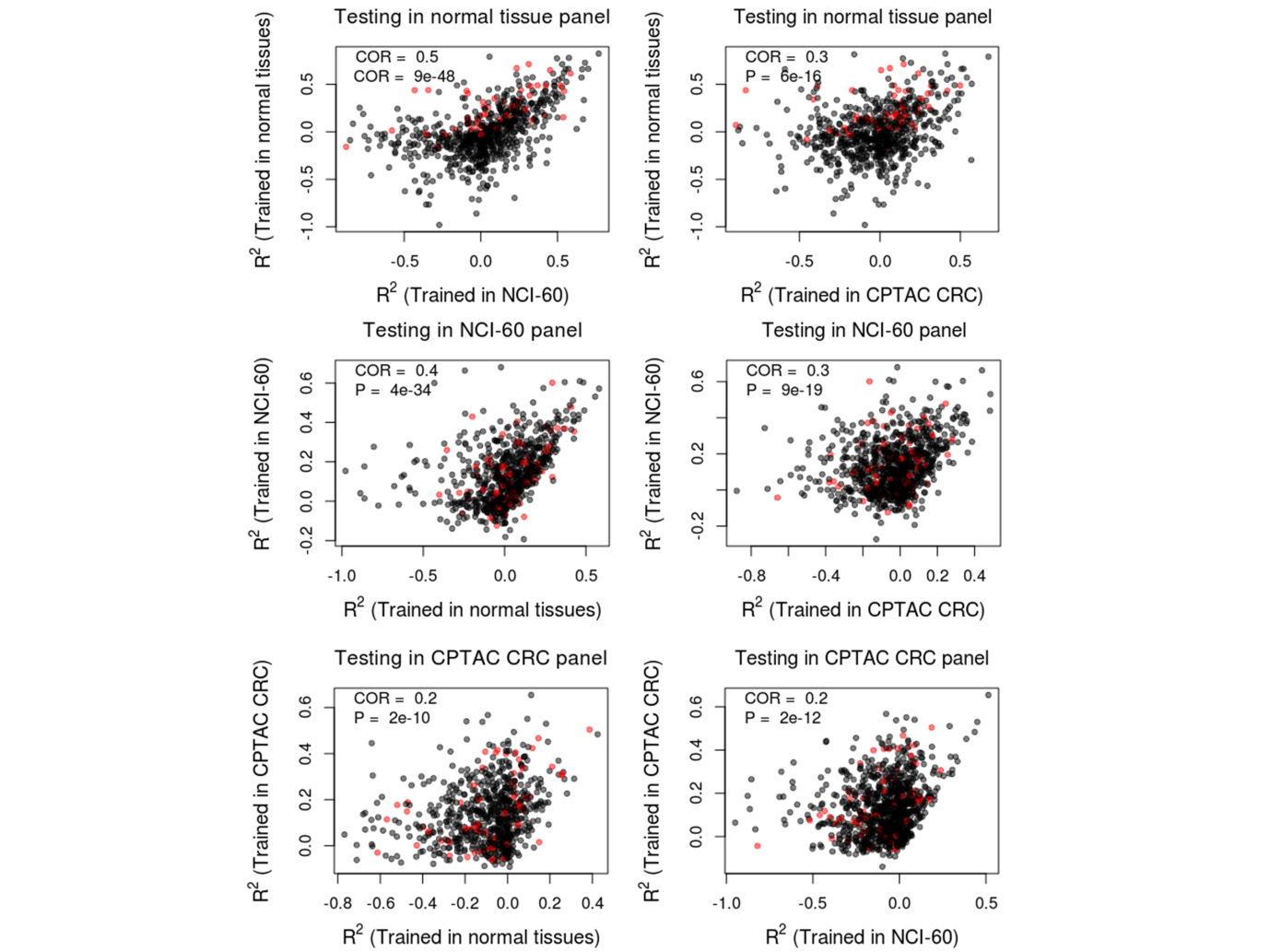


**Supplementary Figure 16** **Cross-panel transferability of models.** RBP^plus^ models show some transferability across tissue panels. Better transferability is observed from NCI-60 and CPTAC CRC panels to normal tissue panel. All the possible combinations of training and test panels are grouped by test panel. Shown is the Spearman’s correlation coefficient between R^2^ of RBP^plus^ models trained in the testing panel (shown in vertical axis label) and R^2^ of RBP^plus^ models trained in the remaining two panels (shown in horizontal axis labels).

**Assessment of alternative translation initiation by ribosomal profiling**

| Ribosomal profiling study | Normal tissue panel | | NCI-60 panel | | CPTAC CRC panel | |
| --- | --- | --- | --- | --- | --- | --- |
|  | Spearman's coefficient | p-value | Spearman's coefficient | p-value | Spearman's coefficient | p-value |
| GSE31539 | 0.02 | 0.5 | -0.016 | 0.8 | -0.011 | 1.0 |
| GSE37744 | 0.19 | 4E-14 | 0.054 | 0.4 | 0.032 | 0.8 |
| GSE41605 | 0.17 | 5E-13 | 0.013 | 0.8 | 0.002 | 1.0 |
| GSE45833 | 0.17 | 5E-13 | 0.052 | 0.4 | -0.008 | 1.0 |
| GSE48933 | 0.19 | 6E-14 | 0.055 | 0.4 | -0.021 | 0.9 |
| GSE55195 | 0.15 | 1E-09 | 0.030 | 0.5 | -0.002 | 1.0 |
| GSE60040 | 0.18 | 2E-13 | 0.046 | 0.4 | -0.003 | 1.0 |
| GSE60095 | 0.15 | 1E-09 | 0.032 | 0.5 | 0.031 | 0.8 |
| GSE61012 | 0.19 | 4E-14 | 0.056 | 0.4 | 0.031 | 0.8 |
| GSE65778 | 0.18 | 4E-14 | 0.037 | 0.4 | 0.033 | 0.8 |
| GSE65912 | 0.11 | 6E-06 | 0.037 | 0.4 | 0.016 | 1.0 |
| SRA056377 | 0.20 | 7E-16 | 0.049 | 0.4 | 0.028 | 0.8 |
| SRA061778 | 0.18 | 4E-14 | 0.010 | 0.8 | 0.037 | 0.8 |

**Table B.** The table reports the Spearman’s correlation between the improvements in accuracy of predicted protein abundance achieved by the RBP^plus^ models relative to the RNA^only^ models and the 5’ UTR-based RPKM values of the mRNAs modelled in the normal tissue, NCI-60 and CPTAC CRC panels. Shown P-values are adjusted by the Benjamini-Hochberg method for controlling false discovery rate at 5%.

**References**

1. [Neuhauser N, Nagaraj N, McHardy P, Zanivan S, Scheltema R, Cox J, et al. High performance computational analysis of large-scale proteome data sets to assess incremental contribution to coverage of the human genome. J Proteome Res. 2013 Jun 7;12(6):2858–68.](http://paperpile.com/b/zJlF6B/sT27)

2. [Altelaar AFM, Munoz J, Heck AJR. Next-generation proteomics: towards an integrative view of proteome dynamics. Nat Rev Genet. 2013 Jan;14(1):35–48.](http://paperpile.com/b/zJlF6B/tVNe)

3. [Bischoff R, Permentier H, Guryev V, Horvatovich P. Genomic variability and protein species - Improving sequence coverage for proteogenomics. J Proteomics [Internet]. 2015 Sep 21; Available from:](http://paperpile.com/b/zJlF6B/YTM2) <http://dx.doi.org/10.1016/j.jprot.2015.09.021>

4. [Lecca P, Re A. Detecting modules in biological networks by edge weight clustering and entropy significance. Front Genet. 2015 Aug 27;6:265.](http://paperpile.com/b/zJlF6B/yflc)

5. [Subramanian A, Tamayo P, Mootha VK, Mukherjee S, Ebert BL, Gillette MA, et al. Gene set enrichment analysis: a knowledge-based approach for interpreting genome-wide expression profiles. Proc Natl Acad Sci U S A. 2005 Oct 25;102(43):15545–50.](http://paperpile.com/b/zJlF6B/gxCng)
